# *Paramecium* BBS genes are key to presence of channels in Cilia

**DOI:** 10.1186/2046-2530-1-16

**Published:** 2012-09-03

**Authors:** Megan Smith Valentine, Anbazhagan Rajendran, Junji Yano, S Dilhan Weeraratne, Janine Beisson, Jean Cohen, France Koll, Judith Van Houten

**Affiliations:** 1 Department of Biology, University of Vermont, 109 Carrigan Drive, Burlington, VT, 05405, USA; 2 Harvard Medical School and Beth Israel Deaconess Medical Center, Boston, 02215, USA; 3 Harvard Medical School and Children’s Hospital, Boston, 02115, USA; 4 Center for Molecular Genetics, National Center for Scientific Research, Génétique Moléculaire, CNRS, Gif-sur-Yvette, 91198, France; 5 Université Paris-Sud, Orsay, 91405, France

## Abstract

**Background:**

Changes in genes coding for ciliary proteins contribute to complex human syndromes called ciliopathies, such as Bardet-Biedl Syndrome (BBS). We used the model organism *Paramecium* to focus on ciliary ion channels that affect the beat form and sensory function of motile cilia and evaluate the effects of perturbing BBS proteins on these channels.

**Methods:**

We used immunoprecipitations and mass spectrometry to explore whether *Paramecium* proteins interact as in mammalian cells. We used RNA interference (RNAi) and swimming behavior assays to examine the effects of BBS depletion on ciliary ion channels that control ciliary beating. Combining RNA interference and epitope tagging, we examined the effects of BBS depletion of BBS 7, 8 and 9 on the location of three channels and a chemoreceptor in cilia.

**Results:**

We found 10 orthologs of 8 *BBS* genes in *P. tetraurelia*. BBS1, 2, 4, 5, 7, 8 and 9 co-immunoprecipitate. While RNAi reduction of *BBS* 7 and *9* gene products caused loss and shortening of cilia, RNAi for all *BBS* genes except *BBS2* affected patterns of ciliary motility that are governed by ciliary ion channels. Swimming behavior assays pointed to loss of ciliary K^+^ channel function. Combining RNAi and epitope tagged ciliary proteins we demonstrated that a calcium activated K^+^ channel was no longer located in the cilia upon depletion of *BBS 7*, *8* or *9*, consistent with the cells’ swimming behavior. The TRPP channel PKD2 was also lost from the cilia. In contrast, the ciliary voltage gated calcium channel was unaffected by BBS depletion, consistent with behavioral assays. The ciliary location of a chemoreceptor for folate was similarly unperturbed by the depletion of *BBS 7*, *8* or *9*.

**Conclusions:**

The co-immunoprecipitation of BBS 1,2,4,5,7,8, and 9 suggests a complex of BBS proteins. RNAi for *BBS 7*, *8* or *9* gene products causes the selective loss of K^+^ and PKD2 channels from the cilia while the critical voltage gated calcium channel and a peripheral receptor protein remain undisturbed. These channels govern ciliary beating and sensory function. Importantly, in *P. tetraurelia* we can combine studies of ciliopathy protein function with behavior and location and control of ciliary channels.

## Background

Cilia and flagella are highly conserved eukaryotic organelles that protrude from the cell surface and whose microtubular axoneme, bounded by a specialized membrane, is assembled from a centriolar structure called the basal body. A variety of sensory functions of cilia have been described in *Chlamydomonas*, *Caenorhabditis* neurons, and epithelial cells, among other cell types. Cilia mediate mechanosensory, chemosensory and photosensory transduction [1-3]. In general, non-motile cilia lack the central pair of microtubules in the axoneme but there are exceptions to this rule, and both motile and non-motile cilia can serve sensory functions [2-7]. The sensory function of motile cilia, although known for a century [8,9], has recently received new attention with the study of sensory aspects of the human motile cilia of the respiratory track [7] and the *Chlamydomonas* flagellum [10].

In humans, the dysfunction of cilia causes severe pleiotropic syndromes known as ciliopathies, that affect a wide variety of tissues, organs and developmental processes [2,4,5,11]. The ciliopathy Bardet-Biedl Syndrome (BBS) is associated with fourteen *BBS* genes and is characterized by a constellation of symptoms: obesity, hypogonadism, polydactyly, retinal degeneration, mental retardation, and kidney cysts [12].

Seven of the fourteen BBS proteins (BBS1, 2, 4, 5, 7, 8, 9) with BBIP10 form the BBSome, which, with the small GTPase Rab8 and its exchange factor Rabin8, is vital for trafficking of Golgi vesicles to the ciliary transport apparatus (intraflagellar transport, IFT) for ciliogenesis [13-16]. Among the ciliary proteins dependent upon the BBSome for trafficking to the cilia are those involved in sensory signaling, such as G protein-coupled and other receptors [16-20]. The BBSome is recruited to the membrane by BBS3 (Arl6) where it forms a coat complex that, with the IFT apparatus, facilitates the incorporation of proteins like the somatostatin receptor into the ciliary membrane [16]. BBS6, 10, 11 and 12 (none of which are in the BBSome) are found in mammals but not in *Chlamydomonas reinhardtii, Caenorhabditis elegans, or Drosophila melanogaster.* BBS6, 10 and 12 appear to form a complex with the chaperonins that are responsible for BBSome assembly [21] (see [11,12,15,22] for reviews).

BBS proteins have been found to play essential roles in non-motile cilia, such as those in olfactory epithelia and primary sensory cilia [19,20], and in motile cilia [6,23]. However, much less is known about the role of BBS proteins in the motile cilia of *Paramecium tetraurelia,* a ciliate covered with thousands of cilia that are responsible for feeding, swimming, and sensory functions. Defects in *Paramecium* ciliary sensory function can be detected by modification of the swimming behavior that depends on the ciliary wave form, which is under electrical control [24,25]. It has been well established that this control is exerted through the activity of ion channels and receptors, many of which reside on the cilia, and respond to environmental signals including touch, chemical food cues, ionic stimuli, and pH [24-27] by fast forward or backward swimming.

In this work, after identification of *P. tetraurelia BBS* gene orthologs and evidence of a BBSome, we present the phenotypes elicited by the depletion of the BBS proteins by RNAi. Our observations of the swimming behavior of *BBS*-depleted cells suggest that ciliary K^+^ channels do not function normally. Given the role of the BBSome in other systems in regulating intracellular trafficking, a possible explanation for the K^+^ channel malfunction is its failure to properly locate or be retained in the cilia. We confirmed this hypothesis by showing that RNAi depletion of *BBS7*, *8* or *9* induced mislocalization of a ciliary calcium-activated K^+^ channel whereas the ciliary localization of a voltage gated calcium channel and a folate chemoreceptor was not affected. In addition, we show that in *Paramecium* motile cilia, as in primary cilia, ciliary trafficking of polycystin-2 (PKD2) is dependent upon the BBS protein function [28].

Our contribution to the understanding of the BBS protein function in motile cilia is through the use of a model organism with a behavioral read-out of ciliary ion channels. A prominent finding of this work is that BBS protein function is essential for localization of a selected set of channels to the cilia, which could lead to sensory and motor defects of motile cilia.

## Materials and methods

### Cell cultures

All chemicals used were purchased from Sigma (St. Louis, MO, USA) unless otherwise noted. *P. tetraurelia*, 51-s (sensitive to killer) were maintained as described in Sasner and Van Houten [29].

### RNAi plasmids

In all construct designs, homologues in other organisms were used to find sequences in the *Paramecium* annotated genome using the dedicated database ParameciumDB (http://paramecium.cgm.cnrs-gif.fr/) [30]. Genes with the highest homology were then used to design constructs for RNAi using genomic DNA (Additional file 1: Table S1). All inserts were ligated into the double T7-promoter vector, L4440 (Addgene, Cambridge, MA, USA). Off-target sequences were searched for using the ParameciumDB database (http://paramecium.cgm.cnrs-gif.fr/cgi/alignment/off- target.cgi). We found that both sequences of each paralog pair would be affected by the same RNAi sequence; BBS2 showed one off-target match of 23 nucleotides from a hypothetical protein; otherwise no other gene sequences in the genome would be targeted by our *BBS* RNAi plasmids.

### Reverse transcription-PCR (RT-PCR)

This method was used as a check on the efficacy of the RNAi feeding, according to our procedures in Yano *et al.*, [31]. We consider the data from RT-PCR to be semi-quantitative and certainly not a suitable way to quantify the degree of mRNA reduction by RNAi. These experiments were repeated a minimum of three times. See Additional file 2: Figure S1 for a representative example.

Calmodulin primers were also used in RT-PCR as a check on the methodology. Concentrations of the cDNA used as template were undiluted, and diluted 10 fold, and 100 fold. Calmodulin primers used were 5^′^-CTGAAGCTGAACTTCAAG-3^′^ (forward) and 5^′^-CAGAATGATGGTTTCTAAATGA-3^′^ (reverse).

### RNAi feeding method

We followed the methods for the *BBS* RNAi as previously described (http://paramecium.cgm.cnrs-gif.fr/RNAi/index.php) [30]. Cells were fed HT115 bacteria transformed with the control (L4440) or with L4440 containing the RNAi insert of interest. After 2 h of incubation while shaking at 37°C, HT115 bacteria transformed with the L4440 plasmid or plasmid with *BBS* insert were induced to produce double stranded RNA by adding isopropylthio-β-galactoside (IPTG) (RPI Corp., Mt. Prospect, IL, USA) to a final concentration of 0.125 mg/ml and incubated for an additional 4 h. The induced culture was centrifuged at 3,439 × g for 10 minutes at 4°C (Beckman J2-21 centrifuge, Beckman Coulter, Brea, CA, USA) and the pellet was re-suspended in 100 ml of wheat grass medium. The 51-s *Paramecium* cells were washed in Dryl’s solution (1 mM Na_2_HPO_4_, 1 mM NaH_2_PO_4_, 1.5 mM CaCl_2_, 2 mM Na-citrate, pH 6.8) and approximately 50 to 100 paramecia were added to the induced culture. Additional stigmasterol, ampicillin, and IPTG were also added to the final concentrations of 8 μg/mL, 0.1 mg/mL, and 0.125 mg/mL, respectively. Cultures were maintained at 28°C. When required, additional induced bacteria, stigmasterol, ampicillin, and/or IPTG were added at 24 and 48 h after feeding. All experiments were carried out at 72 h of RNAi feeding.

The RNAi treatment of cells expressing *FLAG-VGCC1c* was somewhat different because large numbers of cells were required to harvest the cilia. Paramecia were fed bacteria for *BBS8* RNAi or control RNAi as above. The expression of double stranded RNA was induced in 500 mL LB medium with the same concentration of IPTG for 4 h. The final pellets of bacteria were re-suspended in 1.5 L of wheat grass medium containing the same concentrations of stigmasterol (8 μg/mL), ampicillin (0.1 mg/mL), and IPTG (0.125 mg/mL). About 10,000 cells expressing *FLAG-VGCC1c* were added to the bacterial cultures with the *BBS8* RNAi or control plasmids. For three consecutive days, induced bacteria and additional IPTG of 0.125 mg/mL were added to keep cells in log phase. Cells were harvested at 96 h of RNAi feeding for the ciliary membrane immunoprecipitation (IP).

### Fluorescence imaging and ciliary measurements

Cells were imaged using the DeltaVision microscope system and SoftWoRx® Pro software (Applied Precision/GE Healthcare, Issaquah, WA, USA). Images were taken using 20×, 60× or 100× oil emersion objectives on an inverted Olympus IX70 microscope with a Photometrics Coolsnap HQ camera (Photometrics, Tucson, AZ, USA). Lenses used were the UPlanApo 20×/0.80 oil; PlanApo 60×/1.40 oil; PlanApo 100×/1.40 oil. Optical z-sections were 0.5 μm thick. For cilia length measurements, the entire course of a curved cilium in different z-sections was traced using the deconvolved images and softWoRx3.3.6 software in multiple segment mode. Care was taken to match up the segments of the cilia that crossed optical sections. Mann–Whitney *U*-tests of both the raw and normalized data were used to determine significant differences, with no differences in the outcomes. These experiments were repeated three times.

### Scanning electron microscopy

We used scanning electron microscopy to examine 200 mL of cells grown for 72 h in RNAi bacteria. Cells were washed twice in Dryl’s solution using a table top centrifuge to remove debris (Damon/IEC Clinical centrifuge, Needham Hts, MA, USA). Pelleted cells were then treated with 1% osmium tetroxide in 10 mM sodium cacodylate for one minute. Cells were again collected by brief centrifugation and immersed immediately in fresh 2% gluteraldehyde in 10 mM sodium cacodylate buffer. After 10 minutes, cells were centrifuged and rinsed in the same buffer for one hour at room temperature (RT). Cells were then collected by brief centrifugation, placed on 13-mm glass cover slips which had been coated with 0.1% poly-L-lysine (high molecular weight) and rinsed in PBS (137 mM NaCl, 2.7 mM KCl, 10.4 mM sodium phosphate dibasic, 1.7 mM potassium phosphate monobasic, pH 7.4). Cells were allowed to settle for 15 minutes and were then rinsed, stacked, and dried at critical point. Cover slips were glued to an aluminum chuck using graphite cement and allowed to dry. The chuck was then sputter coated and imaged using a JEOL 6060 scanning electron microscope (JEOL USA, Inc., Peabody, MA, USA). These experiments were repeated twice.

### Assays of behavior in response to ionic stimuli

All solutions used to test behavior in ionic stimuli contained a base buffer of 1 mM citric acid, 1 mM Ca(OH)_2_, and 1 mM Tris base. Salts were added from 100 mM stock solutions prepared to desired concentrations (see below) and pH was adjusted to 7.0 using 100 mM Tris Base. After 72 h of growth in RNAi bacteria, approximately 200 cells were removed from their culture and allowed to acclimate in resting buffer (4 mM KCl in the base buffer above) for 30 minutes. Individual cells were transferred to testing solutions in glass depression slides and timed for length of backward swimming; 10 to 20 cells were tested per solution. The experiments were repeated 3 to 10 times. The following solutions were used with the base buffer above: 30 mM KCl; 8 mM BaCl_2_; 25 mM TEA with 10 mM NaCl; and 25 mM TEA with 5 mM MgCl_2_. In some cases, backward swimming durations were normalized to the control backward swimming in order to combine data from many *BBS*-depleted lines. Mann–Whitney *U*-tests performed on the raw data and normalized data showed no difference in significance outcomes. These experiments were repeated a minimum of three times; we often used the swimming in TEA solutions with Na^+^ or Mg^2+^ as indicators of whether the RNAi for *BBS7*, *8*, or *9* was working.

### Deciliation and recovery of motility

Cells were deciliated using trituration in an ethanol solution and observed for recovery of motility. After culturing in 100 mL of RNAi bacteria for 72 h, the cells were collected by centrifugation (Damon/IEC Clinical Centrifuge), washed and re-suspended in Dryl’s solution. Cells were centrifuged again and re-suspended in 4 mM potassium chloride (KCl) buffer as for ionic stimulation. Cells that swam upward in the tube were collected after 5 minutes using a Rainin Pipetman (Mettler Toledo, Columbus, OH, USA) and placed in the same KCl buffer. A sample was removed to confirm that all cells were motile. We rapidly added 100% ethanol to the cells for a final concentration of 5%, sampled cells again to determine motility, and began triturating with a Pasteur pipet to sheer off the cilia. After each course of trituration, the cells were examined to determine how many were no longer motile. These experiments were repeated three times.

### Preparing FLAG pPXV plasmid construct for microinjection

We prepared an N-terminal FLAG pPXV plasmid for expression of three FLAG sequences at the N terminus of the BBS proteins, SK1a (GSPATP00031195001) and VGCC1c (GSPATP00017333001) and a C-terminal FLAG pPXV plasmid with three FLAG sequences for PKD2 (GSPATP00005599001). The pPXV plasmid (courtesy of Dr. W. John Haynes, University of Wisconsin, Madison, WI, USA) with the 3× FLAG or 3× FLAG with insert was extracted using the Wizard^TM^ Plus Mini-Prep (Promega, Madison, WI, USA) and linearized with Not I restriction enzyme (New England BioLabs, Inc., Ipswich, MA, USA). The linearized plasmid was purified, re-suspended at a concentration of 5 to 10 μg/μl in sterile H_2_0, and 5 to 9 pl was injected into the macronucleus of approximately 20 wild type cells. Individual cells were placed into depressions containing 500 μL of inoculated culture fluid and allowed to recover and divide at RT for 24 to 48 h in a humidification chamber. From each depression, 5 to 7 cells were removed and placed in 10 mL of inoculated culture fluid. Each depression was maintained as a separate cell line at 15°C and the cells were re-fed by transferring 5 to 7 cells to fresh culture fluid every 4 days. Cell lines were tested for the presence of the plasmid using PCR with extracted genomic DNA as a template.

### Immunostaining and deconvolution microscopy image analysis

Collection of 100 mL of cultured cells by centrifugation and washing in Dryl’s solution was followed by permeabilization in PHEM solution (60 mM piperazine ethanesulfonic acid (PIPES), 25 mM hydroxyethyl piperazineethanesulfonic acid (HEPES), 10 mM ethylene glycol tetraacetic acid (EGTA), 2 mM MgCl_2_ and 0.1% Triton X-100, pH 6.9) and fixation for 60 minutes in freshly made 4% paraformaldehyde in PHEM. The fixed cells were washed three times with blocking buffer (2 mM sodium phosphate monobasic, 8 mM sodium phosphate dibasic, 150 mM sodium chloride, 1% Tween20, 1% BSA, 10 mM EGTA and 2 mM MgCl_2_; pH 7.4) by centrifugation and incubated for 1 h at RT with primary antibodies: monoclonal anti-FLAG M2 (Sigma) and *Tetrahymena* rabbit anti-centrin-1 (gift from Dr Mark Winey, University of Colorado Boulder, Boulder, CO, USA), or rabbit anti-folate binding protein (FBP) [32]; FBP gene *GSPATP00025147001*[GENBANK: AAS57871]. The cells were collected and washed three times by light centrifugation with 1 mL PBS-T (2 mM sodium phosphate monobasic, 8 mM sodium phosphate dibasic, 150 mM sodium chloride, 1% Tween20; pH 7.4) per wash and incubated for 1 h at RT with 100 μL PBS containing 1:10,000 dilution of secondary antibodies: Alexa fluor® 568-labeled goat anti-mouse and Alexa fluor® 488 goat anti-rabbit (Molecular Probes/Invitrogen, Carlsbad, CA, USA). Cells were washed five times with PBS-T solution and suspended in Vectashield® mounting medium (Vector Labs, Burlingame, CA, USA) for imaging using the DeltaVision® restoration microscopy system (Applied Precision/GE Healthcare, Issaquah, WA, USA) (see fluorescence microscopy and cilia lengths). These experiments were repeated at least three times.

### Whole cell extract (WCE) preparation for immunoprecipitation

The WCE protocol was adapted from previous publications [14,33]. Cells expressing FLAG-tagged *BBS8* or *BBS9* or control cells with the pPXV vector were grown in four 1.5 L wheat grass cultures at 22°C. The cells from the cultures were collected once densities were between 8,000 and 12,000 cells per mL. Cells were washed twice in 200 mL HM Buffer (20 mM Maleic Acid, 20 mM Trizma Base, 1 mM EDTA, pH 7.8), once in 200 mL LAP200 Buffer (50 mM HEPES, 200 mM KCl, 1 mM EGTA, 1 mM MgCl_2_, pH 7.8) and then in 100 mL LAP200 with protease inhibitors: 1 mM phenylmethylsulfonyl fluoride, 1 μg/mL leupeptin (RPI Corp., Mt. Prospect, IL, USA) and 1 μg/mL pepstatin A (RPI Corp.) in addition to 100 μL protease inhibitor cocktail. Cells were then homogenized and the protein concentration was determined using a Pierce protein assay (Thermo Scientific/Pierce, Rockford, IL, USA). Equal concentrations of test and control protein were solubilized by adding Triton X-100 to a final concentration of 1%. Cell lysates were rocked on ice at 4°C for one hour and insoluble proteins were removed by centrifugation at 31,000 × g (Beckman J2-21, Beckman Coulter, Brea, CA, USA) for 20 minutes and then 100,000 × g (Beckman L8-80 M Ultracentrifuge, Beckman Coulter, Brea, CA, USA) for 1 h, both at 4°C.

### Immunoprecipitation with anti-FLAG M2 agarose beads

The protocol for IP of WCE was followed as described previously [14] with some modification: 5 to 6 mL of both control and test WCE were clarified by incubating each lysate with 30 μl of Protein A beads (Amersham/GE Healthcare, Pittsburgh, PA, USA). Anti-FLAG M2 agarose beads (Sigma-Aldrich, St. Louis, MO, USA) were prepared by washing eight times in cold LAP200 buffer containing 1% BSA and 1% TritonX-100 [14]. These prepared beads were added to the clarified sample, incubated on ice while rocking for 2 h, and collected by centrifugation (Damon/IEC Clinical Centrifuge). Beads were washed five times in cold LAP200 buffer with 1% TritonX-100 followed by a final wash in cold LAP200 buffer without TritonX-100. An equal volume (30 to 60 μl) of 2× SDS sample buffer (62.5 mM Tris–HCl, 10% w/v glycerol, 2% SDS, 0.01 mg/mL bromophenol blue, pH 6.8) with 3% β-mercaptoethanol (BME) was added and the sample was boiled for five minutes, and centrifuged at 16,000 × g (Eppendorf centrifuge 5424, Hauppauge, NY, USA) for one minute. The supernatant was then loaded and separated by SDS-PAGE on a 7 to 18% gradient SDS gel. BenchMark^TM^ prestained protein ladder (Invitrogen/Life Technologies, Carlsbad, CA, USA) was loaded to ascertain the approximate molecular mass of the resolved protein samples. Experiments were repeated twice.

Whole cilia were isolated following Adoutte *et al.*[34] and a total of 5.3 mg from control or test RNAi-treated cells was used for IP. The whole cilia were re-suspended in membrane buffer (10 mM Tris buffer, 50 mM KCl, 5 mM MgCl_2_, 1 mM EGTA, pH 7.4) with 1% Triton X-114, and then agitated for 1 h at 4°C. After centrifugation at 16,000 × g (Eppendorf centrifuge 5424) for 10 minutes at 4°C, the supernatant was clarified as previously described using protein A beads (Amersham/GE Healthcare). The clarified lysate was centrifuged at 16,000 × g for 10 min at 4°C and the supernatant was incubated with 20 to 30 μl of prepared anti-FLAG M2 beads (Sigma-Aldrich) for 1 h at 4°C. Beads were prepared by washing four times in membrane buffer with 1% Triton X114. Beads were collected by brief centrifugation and washed in membrane buffer with 1% Triton X-114 and 0.1% BSA three times and then in membrane buffer three times. Samples were prepared as in the WCE before separation by SDS-PAGE.

### Western blots

The proteins separated by SDS-PAGE were transferred to BioTrace^TM^ nitrocellulose blotting membrane (PALL Life Sciences, Ann Arbor, MI, USA). Blots were incubated in blocking buffer comprising 0.5 g skim milk powder, 200 μl of Telost fish gelatin, and 300 μl of normal goat serum (Vector Labs) dissolved in 10 ml of TBS-T (15 mM Tris, 140 mM NaCl, 0.1% Tween, pH 7.5)] at RT for 1 h with rocking. Blots were probed using the following primary antibodies: 1:2500 rabbit anti-FLAG M2 or 1:2000 mouse anti-tubulin. Secondary antibody was either alkaline phosphatase (AP)- or horseradish peroxidase (HRP)-conjugated goat-anti-mouse or anti-rabbit at 1:10,000 dilution and developed accordingly (all antibodies from Sigma-Aldrich, St. Louis, MO, USA).

### Silverstained gels and mass spectrometry analysis

After electrophoresis, the SDS-PAGE gel was stained using directions of the FASTSilver^TM^ kit (G-Biosciences, St. Louis, MO, USA). From the BBS8 and 9 IP, eight regions of each of the silver stained gels were removed (see Additional file 3: Figure S2). The same regions were removed from the control lanes and sliced into small pieces. The molecular mass ranges covered the masses of the BBS proteins (88 kd to 38 kd). Before the trypsin digest, gel slices were destained with 500 μL of destain solution (30 mM K_3_Fe(CN)_6_ and 100 mM sodium sulfate, pentahydrate (EMD Chemicals, Billerica, MA, USA)) at RT for 15 minutes with occasional vortexing. Gel slices were each rinsed twice in 1 mL HPLC water for 5 minutes, then were covered in 100 μL 100 mM NH_4_CO_3_. This was removed after incubating at RT for 10 minutes, and samples were incubated at 37°C for 10 minutes in 500 μL 50% MeCN in 50 mM NH_4_CO_3_. This was removed, 500 μL was again added and the samples were incubated at 37°C for 30 minutes. Samples were dehydrated using 100 μL 100% MeCN and incubated at RT for 10 minutes. Samples were spun briefly and the solution removed. Gel pellets were allowed to dry completely and were then incubated in 30 to 60 μL of 12.5 ng/μL trypsin (Promega, Madison, WI, USA) in 50 mM NH_4_CO_3_ at 37°C. After 10 to 15 minutes, samples were given an additional 30 μL of 50 mM NH_4_CO_3_ if needed and then incubated at 37°C overnight. The following morning, the solution was removed and saved. The gel slices were vortexed in 150 μL of 50% MeCN and 2.5% formic acid and spun for 10 minutes at 16,000 × g (Eppendorf centrifuge 5424). The supernatant was removed and added to the trypsin sample removed earlier for each sample. The gel slices were lastly dehydrated in 100 μL of 100% MeCN for 5 minutes at RT, then centrifuged at 16,000 × g for 5 minutes. Each supernatant was again removed and added to the other collected supernatants for each sample. These collected digested peptides were then dried completely in a SpeedVac, re-suspended in 8 μL of 2.5% MeCN and 2.5% formic acid, and 4 μL was transferred to a 100 μL glass deactivated tube with polymer feet (Agilent Technologies, Santa Clara, CA, USA). Each tube was then capped in a glass autosampler vial.

Tryptic peptides in an autosampler vial were loaded using a Micro-autosampler (ThermoElectron, Waltham, MA, USA) onto a microcapillary column packed with 12 cm of reversed-phase MagicC18 material (5 μm, 200 Å; Michrom Bioresources, Inc., Auburn, CA, USA). After a 15-minute isocratic loading at 2.5% MeCN and 0.5% formic acid, peptides were eluted with a 5 to 35% MeCN (0.1% FA) gradient over 60 minutes. Ten mass spectrometry (MS/MS) scans followed each survey scan for the entire run (75 minutes). Mass spectra were acquired in a LTQ-XL linear ion trap mass spectrometer (Thermo Electron).

The raw MS/MS data were searched against the *Paramecium tetraurelia* forward (target) and reverse (decoy) proteome databases (http://aiaia.cgm.cnrs-gif.fr/download/fasta/) [35], using the Sequest algorithm with a precursor mass tolerance of 2 Da. A static increase in 71.0 Da of cysteine residues for acrylamide adduction was required and differential modification of 16.0 Da on methionine residues was permitted. The top matches were filtered using a unique delta correlation score (dCn2) of 0.16 and Xcorr (cross-correlation) values of 1.8, 2.4 and 2.8 for single-, double- and triple-charged ions, respectively. At the protein level, only proteins for which two unique peptides were assigned to a given Genoscope annotation identification were retained. The number of protein entries having an identical peptide match is listed for each peptide in the column ‘redu’ for redundancy. The estimated false-discovery rates were calculated based on the number of reverse database assignments after the above filtering was applied. Specifically the false discovery rate equals the number of peptides assigned to a reverse database entry times two (to account for unknown false positives) divided by the number of peptides assigned to the forward database. The estimated false discovery rate of proteins identified by more than two unique peptides for the BBS8 WCE IP sample was 0.36% and for the BBS9 WCE IP sample was < 0.00%. The tryptic peptides were analyzed twice for each BBS IP.

## Results

### *Paramecium BBS* genes

A search of the *P. tetraurelia* genome identified eight *BBS* homologs (*BBS1, 2, 3, 4, 5, 7, 8* and *9*) (Additional file 1: Table S1). *BBS3* and *BBS5* are each encoded by two paralogs, originating from genome-wide duplications [35]. The *BBS3aBBS3b* and *BBS5aBBS5b* pairs share 85% and 86% nucleotide identity respectively, a sequence conservation that allows co-inactivation of the paralog when one gene is silenced by RNAi (see Materials and Methods).

#### *BBS protein interactions in Paramecium*

**Figure 1:**
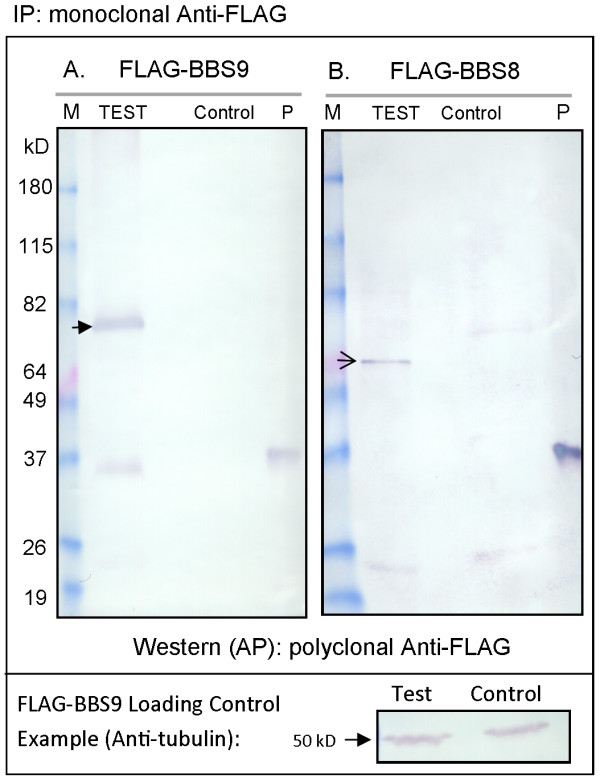
**Immunoprecipitation (IP) of FLAG-BBS9 or FLAG-BBS8 proteins from whole cell extracts (WCEs). **Proteins were immunoprecipitated using anti-FLAG affinity beads and western blots were developed (AP) using polyclonal anti-FLAG. **(A)** IP from cells expressing *FLAG-BBS9* (Test) and control cells expressing the FLAG plasmid (Control). Closed arrow indicates FLAG-BBS9. **(B)** IP from cells expressing *FLAG-BBS8* (Test) and control cells expressing the FLAG plasmid (Control). Open arrow indicates FLAG-BBS8. Both blots show a molecular weight marker (M) and a 37 kDa FLAG-fusion protein as a positive control (P). Below is an example of a loading control from the FLAG-BBS9 IP. Protein concentrations were determined using a Pierce assay before solubilization to ensure equal amounts of protein were used for both the Test and Contol IP. The loading control blot was probed with anti-tubulin (50 kD).

In order to determine the BBS protein interactions in *P. tetraurelia*, we expressed *FLAG*-tagged *BBS8* and *BBS9* genes separately and analyzed the proteins immunoprecipitated from WCEs after separation by SDS gel electrophoresis. Control cells expressed the empty FLAG vector. The presence of FLAG-BBS8 or -BBS9 was confirmed using western blotting (Figure 1) compared to the control cells. We do not know the identities of the bands below 37 kD in the test lanes. The positive control lanes in Figure 1A and B (labeled P) show the reactivity of a small FLAG-fusion protein.

IPs from WCEs of control cells and those transformed with *FLAG-BBS8* or *-BBS9* expression vectors were separated by SDS gel electrophoresis and were silver stained (Additional file 3: Figure S2). The gel lanes were divided into eight sections that spanned molecular masses from 38 to 88 kD, treated with trypsin, and analyzed by tandem MS/MS (for details, see Materials and Methods). We considered peptides found only in the lanes from the FLAG-BBS IPs and not in the control lanes. Proteins were identified by comparison with the *Paramecium* annotated genome. We required each protein positively identified to have two or more peptides and for these peptides to be absent from the control (Table 1). Using these methods, in IPs from cells expressing *FLAG-BBS9*, we found BBS1, 2, 4, 5, 7, and 8 (Table 1a). In IPs from cell expressing *FLAG-BBS8*, we found BBS1, 2, 5, 7, and 9 (Table 1b). Although mammalian BBS4 has been shown to precipitate with BBS8 and BBS9 [14], we failed to identify BBS4 in our FLAG-BBS8 IP experiment, possibly because the FLAG tag interfered with that specific protein interaction, or the interactions were not maintained under our IP conditions. There is no *Paramecium* ortholog of BBSome protein BBIP10 [13] and we did not find a candidate for BBIP10 in our IPs. BBS3 is not part of the mammalian BBSome [14], and did not precipitate with BBS8 or BBS9 in our experiments. Peptides for the specific BBS proteins were found in the molecular mass range expected, indicating no anomalous distribution of peptides due to degradation of the proteins in the gels (Additional file 3: Figure S2). The non-BBS proteins that precipitated with BBS9 or 8 are listed in Additional file 4: Table S2 and Additional file 5: Tables S3.

**Table 1:** Mass Spectrometry Results. BBS proteins co-immunoprecipitated with FLAG-BBS9 (a) or FLAG-BBS8 (b)

**Gene name**	**Annotated gene accession number**	**MW (kD)**	**Peptides identified in FLAG-BBS9 IP (number)**		**Peptides identified in control IP (number)**
			**Unique**	**Total**	**Total**
*BBS9*	GSPATP00027545001	83.33	11	30	0
*BBS7*	GSPATP00026091001	77.44	9	16	0
*BBS2*	GSPATP00000964001	75.48	10	18	0
*BBS1*	GSPATP00033252001	64.85	11	18	0
*BBS8*	GSPATP00028481001	58.61	8	26	0
*BBS4*	GSPATP00005292001	50.07	7	11	0
*BBS5*	GSPATP00036912001	38.19	5	12	0
**Gene name**	**Annotated gene accession number**	**MW (kD)**	**Peptides identified in the FLAG-BBS8 IP (number)**		**Peptides identified in control IP (number)**
			**Unique**	**Total**	**Total**
*BBS8*	GSPATP00028481001	58.61	4	8	0
*BBS9*	GSPATP00027545001	83.33	13	32	0
*BBS7*	GSPATP00026091001	77.44	7	9	0
*BBS2*	GSPATP00000964001	75.48	7	8	0
*BBS1*	GSPATP00033252001	64.85	7	19	0
*BBS5*	GSPATP00036912001	38.19	2	2	0

#### *Ciliary phenotypes of BBS depleted paramecia*

*P. tetraurelia* provides the interesting advantage of monitoring channel activity by observation of swimming behavior. For example, transient backward swimming caused by the reversal of the cilia power stroke correlates with a calcium action potential. Depolarization above threshold initiates a graded Ca^2+^ action potential by opening the voltage-gated Ca^2+^ channels (Ca_v_) that are exclusively in the cilia [24,25,36]. The resulting increase in intra-ciliary Ca^2+^ changes the power stroke of the cilia, sending the cell backward. A rapidly activated voltage-gated K^+^ conductance (I_Kv_) and slower calcium activated K^+^ conductance (I_KCa_) return the membrane potential to resting levels. The K^+^ channels responsible for this conductance are also thought to be in the cilia and the Ca^2+^ that activates the calcium-dependent potassium channel (K_Ca_) is local (not cellular) Ca^2+^, entering cilia through the ciliary Ca_v_ channels [26,37]. The duration of backward swimming is a function of the number and activity of Ca_v_ channels as well as the repolarizing K^+^ conductance. In addition, other Ca^2+^-dependent channels that conduct Na^+^ or Mg^2+^ can prolong the plateau of the action potential, and increase the duration of backward swimming. Since paramecia survive in a range of buffers, we are able to assess the separate contributions of K^+^, Na^+^ and Mg^2+^ conductances to the backward swimming.

Throughout, we refer to cells fed bacteria transformed with the L4440 vector as the control cells and to cells fed bacteria transformed with a vector containing an RNAi insert as the depleted cells. While we have confirmed by RT-PCR that mRNA has been reduced by RNAi (Additional file 2: Figure S1), we assume that the BBS proteins are likewise depleted but not eliminated. (See Additional file 6: Figure S3 for evidence of loss of the FBP with RNAi).

*Paramecium* cells were depleted for each of the BBS proteins individually. Overall examination with a dissecting microscope revealed no anomalies in cell shape, growth rate and swimming speed upon depletion of *BBS1-5* and *BBS8*. A slow swimming speed was observed for *BBS7*- and *BBS9*-depleted cells, which eventually died after 72 h of RNAi.

**Figure 2:**
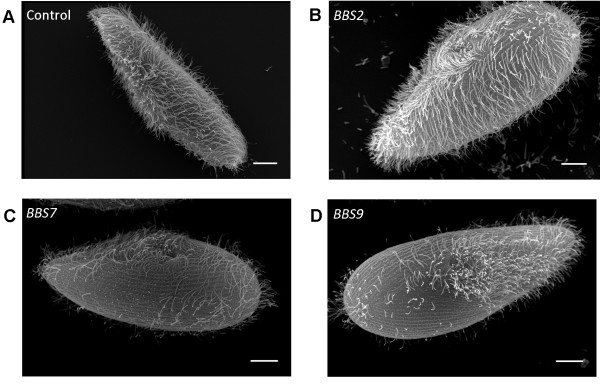
**Scanning electron microscopy images of control and BBS depleted cells. **(**A**) Control cell, (**B**) *BBS2*-depleted cell, (**C**) *BBS7*-depleted cell and (**D**) *BBS9*-depleted cell. Scale bars are 10 μm; these are representative images.

**Figure 3:**
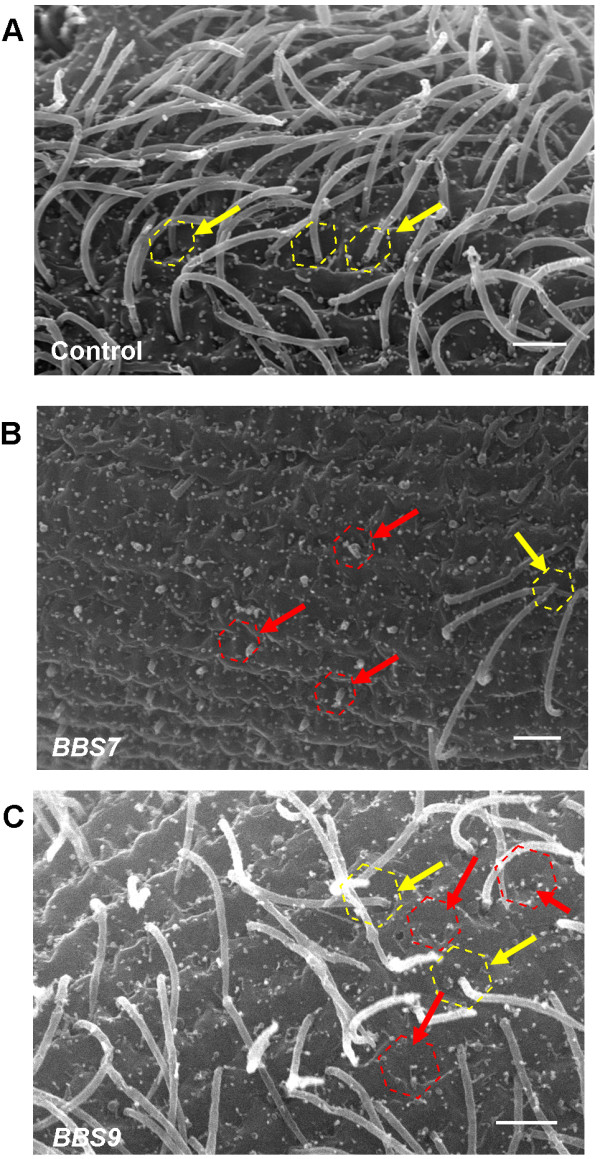
**Scanning electron micrographs of cilia and cell surfaces. **(**A**) Control cell; yellow dotted hexagon and arrows emphasize cortical units with a cilium arising from the center. (**B**) *BBS7*- depleted cell. (**C**) *BBS9*-depleted cell. Red dotted hexagons and red arrows indicate cortical units with short cilia arising, or no cilia. Note these cells contain cortical units with average length cilia arising (yellow dotted hexagons and yellow arrows). These are representative images, scale bars represent 2 μm.

To examine the distribution of cilia, we made scanning electron microscope images of the *BBS*-depleted cell lines. Figure 2 shows a representative selection of cells all of which fall within the normal range of size and shape for cells in an asynchronized culture. The *BBS2*- depleted cell in Figure 2B is representative of *BBS3*-*, BBS4*-*, BBS5*-*,* and *BBS8*-depleted cells, all of which show a distribution of cilia comparable to that of the control cells (Figure 2A). In contrast, the *BBS7*- and *BBS9*-depleted cells (Figures 2C,D) differ dramatically from the control and *BBS2*-depleted cells by showing patches of surface with no cilia and even totally bald cells. Additional file 7: Figure S4 shows the distributions of bald and partially ciliated cells for *BBS7*-, *BBS8*-, and *BBS9*-depleted cells. Figure 3A shows more detail of control normal cilia and surface and the normal surfaces with stubs of cilia, short cilia and bald patches of *BBS7*- and *BBS9*-depleted cells (Figure 3B,C).

**Figure 4:**
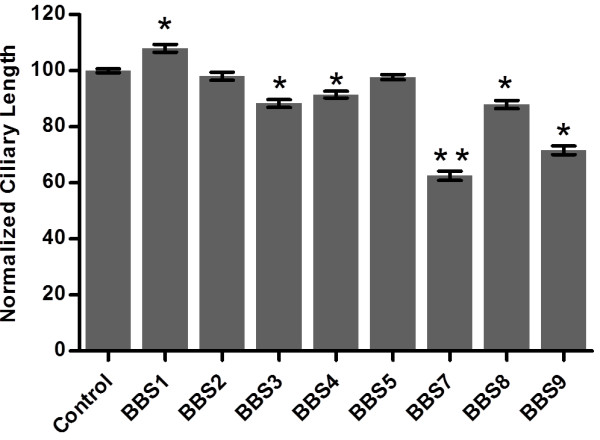
**Normalized ciliary lengths of *BBS*-depleted cells. **Data are averages of 138 to 798 cilia on 15 to 66 cells from three replicate experiments ± standard error of the mean (SEM). The control value before normalization was 11.72 μm ± 0.08 μm (average ± SEM, n = 798). Significant changes determined by the Mann–Whitney *U*-test are denoted as *P < 0.05, **P < 0.0001.

We examined ciliary length using anti-tubulin antibodies for immunofluorescence imaging (Figure 4). The RNAi feeding had little effect on ciliary length with the exception of *BBS7* and *BBS9* depletion. The length of cilia in other *BBS*-depleted cell lines range from 88% to 108% of controls, while the few cilia that remained on the *BBS7*- and *BBS9*-depleted cells were 62% and 72% of the control length, respectively.

To be sure that bald patches on *BBS7*- and *BBS9*-depleted cells were not just due to more fragile cilia than in the control cells and therefore more easily lost during fixation, we assayed their deciliation capacity by trituration in 5% ethanol (see Materials and Methods). Deciliation required as many trituations of the *BBS7*- and *BBS9*-depleted cells as control cells to render the cells non-motile: 10 triturations immobilized 52% of both control and *BBS7*-depleted cells and 63% of both control and *BBS9*-depleted cells.

#### *Behavioral phenotypes of BBS-depleted paramecia*

**Figure 5:**
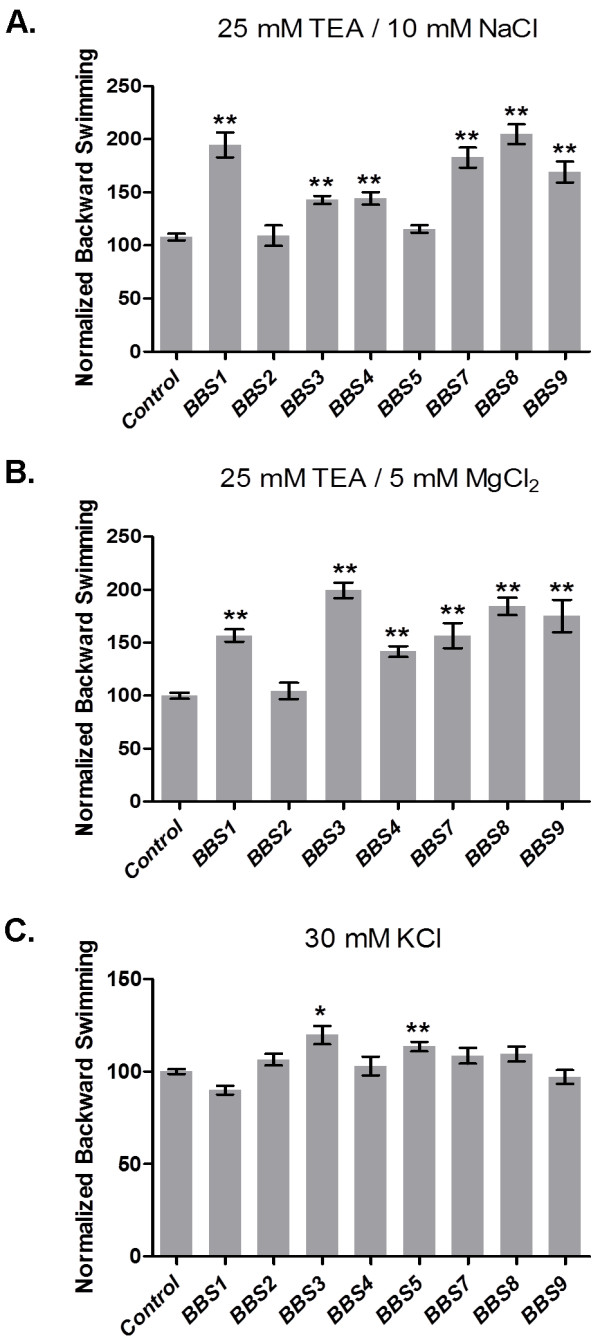
**Backward swimming duration after depolarization in tetraethylammonium chloride (TEA) with Na**^**+**^**, TEA with Mg**^**2+**^**, or KCl. **Durations of backward swimming (sec) in buffers with: (**A**) 25 mM TEA with 10 mM NaCl, (**B**) 25 mM TEA with 5 mM MgCl_2;_ and (**C**) 30 mM KCl. Data are normalized to the control backward swimming duration. Asterisks denote significant difference from control using the Mann–Whitney *U*-test (*P < 0.05; **P < 0.0001). Data are averages from three experiments; n = 45 to 418 (**A**); 45 to 165 cells (**B**) and 25 to 240 (**C**). Data are averages ± standard error of the mean (SEM).

In order to assess the sensory function of the cilia on the RNAi-treated cells, we analyzed swimming behavior by measuring the duration of backward swimming in a battery of depolarizing solutions (see Materials and Methods). With the exception of *BBS2*-depleted cells, which show normal behavior in all the testing solutions, *BBS*-depleted cells show prolonged backward swimming both in TEA solutions with Na^+^ (Figure 5A) and TEA solutions with Mg^2+^ (Figure 5B) (*BBS1*, *3*, *4*, *7*–*9*). These behaviors are consistent with a reduced I_K(Ca)_ of the cilia.

TEA inhibits the voltage-dependent potassium channels (K_v_), allowing us to focus on the remaining K_Ca_ above. To examine the function of K_v,_ we employed 30 mM KCl. Only *BBS3*-and *BBS5*-depleted lines showed slightly but significantly longer backward swimming in 30 mM KCl (Figure 5C). Given that all the *BBS*-depleted cells including *BBS3* and *BBS5* show normal behavior in 8 mM BaCl_2_ (Additional file 8: Table S4), we presume that their Ca_v_ channels are operating normally, and that a reduced I_Kv_ is causing a prolonged backward motion of *BBS3*- and *BBS5*-depleted cells in 30 mM KCl.

It should be noted that while *BBS7* and *BBS9* depleted cells swim more slowly due to the loss of cilia, their backward swimming durations in solutions other than those with TEA were normal. That is, the short or lost cilia do not create a general long backward swimming phenotype in all depolarizing solutions, but rather the cells show a phenotype in TEA with Mg^2+^ and TEA with Na^+^ that is consistent with a loss of specific K_Ca_ channels.

#### *Effect of BBS depletion on ciliary membrane proteins*

**Figure 6:**
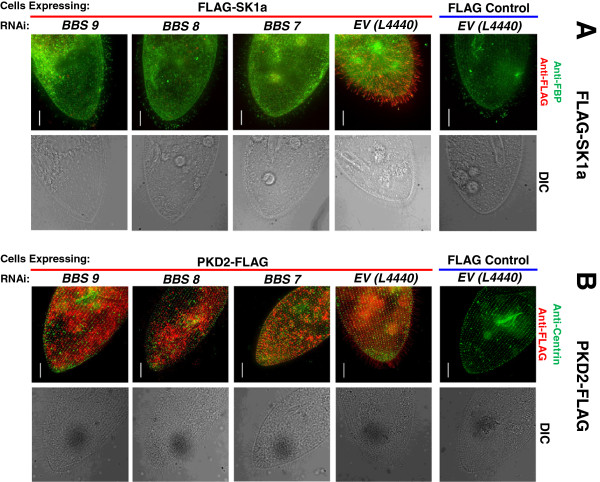
**Immunofluorescence of FLAG-SK1a channels or PKD2-FLAG channels in *BBS7 *- or *BBS8 *- or *BBS9*-depleted cells). **(**A**) FLAG-SK1a channels. (**B**) PKD2-FLAG channels. *FLAG-SK1a*- expressing cells were stained with anti-FLAG antibody and as a contrast, anti-folate chemoreceptor (FBP) antibody in a series of control and RNAi conditions. Only the merged images are shown here. Complete immunofluorescence of the folate chemoreceptor and the FLAG-SK1a channel are shown separately in Additional file 9: Figure S5. The FLAG control is a cell microinjected with FLAG-pPXV vector and fed with RNAi empty vector (L4440) bacteria. The FLAG-SK1a control is a cell expressing *FLAG-SK1a* and fed bacteria with an RNAi empty vector (L4440). *BBS7*, *BBS8* and *BBS9* are the cells expressing FLAG-SK1a channel and also *BBS7*-, *BBS8*- or *BBS9*-depleted, respectively. Cells were immunostained with anti-FLAG (red) and anti-FBP (green) antibodies. Differential interference contrast (DIC) images are shown to document that cilia are present. Cells expressing *PKD2-FLAG* channel (**B**) were stained with anti-FLAG antibody (red) and anti-*Tetrahymena* centrin-1 antibody (green) in a series of control and RNAi conditions. Only the merged images are shown here; the staining of FLAG and centrin can be seen separately in Additional file 10: Figure S6. The FLAG control is a cell expressing the FLAG-pPXV vector and fed with RNAi empty vector (L4440) bacteria. The PKD2-FLAG control is a cell expressing the *PKD2-FLAG* channel and fed with bacteria with RNAi empty vector (L4440). *BBS7, BBS8* and *BBS9* are the cells expressing the *PKD2-FLAG* channel and are *BBS7*-, *BBS8*- or *BBS9*- depleted, respectively. Differential interference contrast (DIC) images are shown to document that cilia are present. All images were taken under 60× oil immersion objectives. Scales represent 15 μm. Images are representative of results of three experiments, n = 126 to 156 cells.

**Figure 7:**
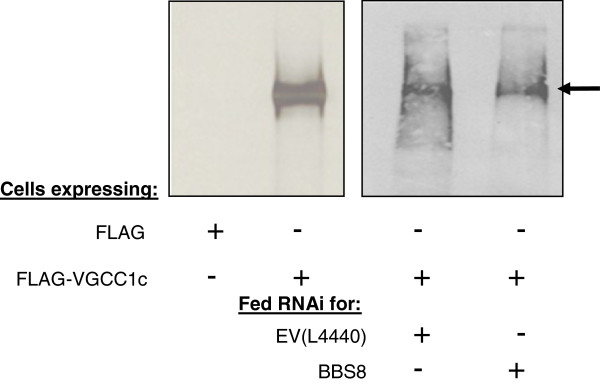
**Immunoblots (ECL) of proteins immunoprecipitated from ciliary membranes. **Ciliary membranes were solubilized in Triton X-114 and the proteins were immunoprecipitated using anti-FLAG affinity beads and separated on a 5-18% SDS-PAGE gel. (**A**) Control experiment where cells were expressing the empty FLAG vector (control) or *FLAG-VGCC1c* (TEST). The IP of the FLAG-VGCC1c can be seen in the TEST lane (arrow: 272 kD). (**B**) Cells expressing *FLAG-VGCC1c* were fed bacteria with the *BBS8* RNAi plasmid *(BBS8)* or the empty RNAi vector (EV L4440) as a control. The immunoblots were developed using polyclonal anti-FLAG. Arrow indicates the band of the FLAG-VGCC1c proteins.

In the behavioral experiments, all the *BBS*-depleted cells except *BBS2* show phenotypes consistent with reduced function of K_Ca_, K_v_, or both K_Ca_ and K_v_. One possible reason for the failure of ciliary K^+^ channels to function in *BBS*-depleted cells is that they are no longer located in the cilia. In order to test this possibility, we expressed a FLAG-tagged small conductance potassium channel (SK1a) that is a member of the K_Ca_ channels. We use this SK1a as a proxy for the two K_Ca_ channels of the cilia. We also examined cilia for the presence of PKD2 and voltage-dependent calcium channel isoform 1c (VGCC1c), a Ca_v_ that is exclusively in the cilia. We chose these channels because we know from proteomics studies and epitope-tag expression studies that they are expressed in *P. tetraurelia* cilia [38]. Figure 6 (and Additional file 9: Figure S5, Additional file 10: Figure S6) show the results of these experiments using cells transformed with vectors for expression of FLAG alone or FLAG-tagged channels combined with separate RNAi treatments for each of three *BBS* orthologs, *BBS7, 8,* or *9.* We chose *BBS8* because its depletion leads to the same apparent loss of K_Ca_ channel function as *BBS7* and *BBS9* depletions while having very little effect on cilia number or length. We chose *BBS7* and *BBS9* because they caused dramatic phenotypes in cilia number and length when depleted. We used immunofluorescence to examine SK1a and PKD2 only on cells that retained cilia as shown in the differential interference contrast (DIC) images (Figure 6). For VGCC1c, we examined western blots of IPs of ciliary membrane because of the low expression of this channel in the cilia (Figure 7).

Before immunostaining of the cells (Figure 6 and Additional file 9: Figure S5 and Additional file 10: Figure S6), we confirmed that the *BBS7, 8* or *9* depletion was effective by assaying the cell behavior in solutions of TEA with Mg^2+^ and TEA with Na^+^ (see Additional file 11: Figure S7). The data from three replicate experiments show that the RNAi treatments result in significantly longer backward swimming in these solutions, while the control cells show normal durations of backward swimming.

There are two control conditions for each experiment shown in Figure 6 (and Additional file 9: Figure S5 and Additional file 10: Figure S6) with FLAG-SK1a and PKD2-FLAG: paramecia transformed with the expression vector with FLAG, and fed bacteria with the control RNAi vector (FLAG Control) and paramecia expressing the FLAG-tagged *SK1a* or *PKD2* channels and fed bacteria with the control RNAi vector (Empty RNAi vector Control). The test cells expressing FLAG-tagged *SK1a* or *PKD2* were fed RNAi bacteria to deplete *BBS7, 8* or *9* (Figure 6, *BBS7, BBS8* and *BBS9).* In the first column of images, we used monoclonal anti-FLAG antibody to detect FLAG-SK1a (red) and as a contrast (green) an antibody against a folate chemoreceptor that is known to be in the cilia (anti-FBP). The corresponding DIC images are shown to assure that the cells were ciliated. In the second column of fluorescence images, we used the monoclonal anti-FLAG antibody to detect PKD2-FLAG (red) and a polyclonal anti-Centrin1 (green) that is specific for basal bodies so that the surface structure would be apparent. In Figure 6 we show only the merged fluorescence and DIC images; the separate images can be found in Additional file 9: Figure S5 and Additional file 10: Figure S6.

In both sets of experiments for FLAG-SK1a and PKD2-FLAG in Figure 6, the tagged channels are clearly elaborated in the cilia (Empty RNAi Vector Control) and are no longer found in the cilia when *BBS7, BBS8* or *BBS9* is depleted. Notably, the folate chemoreceptor remains on the cilia, especially at the tips, even though the FLAG-SK1a channel is no longer evident in the cilia with *BBS* depletion. The DIC images demonstrate that we have chosen cells that retain cilia and matched them for region of the cell and dorsal vs. ventral side. In this way, we demonstrate that the loss of FLAG-SK1a or PKD2-FLAG staining is not due to lack of cilia.

As a measure of the effectiveness of our RNAi technique in reducing protein levels, we repeated the experiment with FLAG-SK1a expression in *BBS7*-*, BBS8*- or *BBS9*-depleted cells, and concurrently used RNAi feeding to reduce the folate chemoreceptor as well. As shown in Additional file 6: Figure S3, we see the same results for loss of SK1a in the cilia of *BBS7*-*, BBS8*- or *BBS9*-depleted cells as in Figure 6 and also greatly diminished folate chemoreceptor detected by the anti-FBP antibody.

The behavioral tests predicted that the Ca_v_s, which are exclusively in the cilia, are present and functional in *BBS7, BBS8*- and *BBS9*-depleted cells. In order to confirm this, we expressed the full length FLAG-tagged sequence for one of the Ca_v_s (VGCC1c) that we have found in the cilia by MS/MS [38]. This channel is in very low abundance, which made it necessary to use IP of ciliary membrane and immunoblotting rather than immunofluorescence. Figure 7 shows a representative blot of anti-FLAG reactive proteins from ciliary membrane of control cells and those depleted for *BBS8*. Since we must harvest large amounts of cilia for this experiment, it could not be repeated on *BBS7*- or *BBS9*-depleted cells. The FLAG-VGCC1c band of about 272 kD is present in both control and depleted cells. As for the experiments in Figure 6, the efficacy of the RNAi was monitored by the duration of backward swimming in TEA-Na solutions (Additional file 11: Figure S7).

## Discussion

The distinct distributions of ion channels in the plasma membrane and cilia of *Paramecium* provide a different window to observe the role of BBS proteins in trafficking of channels and other ciliary membrane proteins. Deciliation and reciliation studies have demonstrated that Ca_v_ channels reside exclusively in the *Paramecium* cilia and that these channels are not evenly distributed along the cilium, with fewer at the proximal end of the cilia [25,36]. The voltage-gated and calcium-activated K^+^ channels (K_v_ and K_Ca_) are also found in the cilia and, like the Ca_v_ channels, might be concentrated there [26]. The Ca^2+^ that activates the K_Ca_ channel has been shown to come from the Ca_v_ channels of the cilia [37]. Since there is no spill-over of Ca^2+^ from action potentials from cilia to cell body, K_Ca_ channels that repolarize after the action potential appear to reside in the cilia in order to be activated [39]. Given this uneven distribution of channels between the cilia and cell body plasma membrane, the location of channels in the cilia vs. plasma membrane is key in maintaining the cells’ normal responses to many ionic stimuli. Our RNAi studies for *BBS7, BBS8,* and *BBS9* in cells expressing epitope-tagged *SK1a*, *VGCC1c* and *PKD2* channels suggest that these BBS proteins have a role in this distribution. The SK1a and PKD2 channels are not present in the cilia of the RNAi-treated cells, while the VGCC1c channel appear to be present.

Except for BBS2, the behaviors of the *BBS*-depleted cells suggest a *BBS* phenotype that is consistent with our physical evidence of loss of K^+^ channels and retention of VGCC1c in cilia of cells treated with *BBS7, BBS8*, and *BBS9* RNAi. More specifically, the *BBS* RNAi phenotype behavior is characterized by prolonged backward swimming in specific depolarizing solutions and consistent with loss or reduction of function of the K_Ca_ channel or K_v_ channel or both (Table 2). TEA inhibits the K_v_ channel [40], leaving the K_Ca_ channels to repolarize the cell after an action potential and end backward swimming. Because *BBS1*-, *3*-, *4*-, *7*-, *8*-, and *9*-depleted cells swim backward much longer than control cells in both Mg/TEA and Na/TEA solutions, there should be an ineffective I_KCa_ conductance rather than prolonged Na^+^ or Mg^2+^ inward conductance in the depleted cells. *BBS3*- and *BBS5*-depleted cells also show backward swimming that is slightly but significantly longer than control in 30 mM KCl, indicating that the ciliary K_v_ channels do not efficiently repolarize the cells following a depolarizing stimulus of high extracellular K^+^. Therefore, we propose that *BBS3*-depleted cells have defects in both K_v_ and K_Ca_ channel function and that *BBS5*-depleted cells have defects in the K_v_ channel function only. The Ca_v_ channel is not responsible for the prolonged backward swimming in K^+^ or TEA solutions in any of the BBS phenotypes, since prolonged opening of the Ca_v_ channels was not evident as longer backward swimming in other depolarizing stimuli like Ba^2+^ that is specific for the Ca_v_ (Additional file 8: Table S4).

**Table 2:** Summary of BBS phenotypes

**RNAi**	**Changes in cilia length**	**Long backward swimming in 30 mM KCl**	**Long backward swimming in Na**^**+**^**and Mg**^**2+**^**TEA solutions**	**Disrupted conductance**
*BBS1*	Longer	No	√	I_K(Ca)_
*BBS2*	No	No	No	none
*BBS3*	Short	√	√	I_K(Ca)_, I_K(V)_
*BBS4*	Short	No	√	I_K(Ca)_
*BBS5*	No	√	No	I_K(V)_
*BBS7*	Short and missing	No	√	I_K(Ca)_
*BBS8*	Short	No	√	I_K(Ca)_
*BBS9*	Short and missing	No	√	I_K(Ca)_

Interestingly, even though *BBS7*- and *BBS9*-depleted cells have fewer and shorter cilia, their cilia seem to have selectively reduced or lost K_Ca_ channel function and not Ca_v_ function. Importantly, the loss of K^+^ channel function is not merely a side effect of the shortening or loss of cilia since *BBS3*-, *BBS4*-, *BBS5*-, and *BBS8*-depleted cells with full or almost full-length cilia show this BBS phenotype of selective loss of K^+^ channel function.

Our co-IP studies of *P. tetraurelia BBS8* and *BBS9* (Table 1) suggest that there is a complex of BBS proteins that has the same composition as the BBSome in mammals [14]. The Meckel-Gruber Syndrome 1 (*MKS1*) gene has only recently been identified as *BBS13*[41] and does have an ortholog in *P. tetraurelia.* BBS13 is not part of the BBSome [14] and has not been included in this study*.* Also, there is no clear ortholog for the BBSome associated protein BBIP10.

Similar to SK1a, the PKD2 channel was also missing from the cilia of the *BBS7, BBS8*- and *BBS9*-depleted cells, but the loss of this channel from the cilia does not explain the K^+^ channel-specific BBS phenotype. Indeed, PKD2 depletion produces a phenotype consistent with the loss of the Mg^2+^ channel function, that is, short backward swimming in Mg^2+^ solutions, and not with a loss of K^+^ channels (Valentine, personal communication). As is evident in Figure 6, PKD2 is found on both the cilia and the cell surface, and we surmise that its absence from the cilia in *BBS*-depleted cells does not affect its function in Mg^2+^ conductance. As in mammalian cells, *Paramecium* PKD2 is dependent upon BBS proteins for its proper location in the ciliary membrane [28]. In *Caenorhabditis elegans*, there are additional cell type-specific mechanisms for PKD2 to reach the cilia [42], including interactions with PKD1 that has no ortholog in *P. tetraurelia*. The PKD2 of *Chlamydomonas* is retained in the flagella of *bbs4* mutants, and other proteins accumulate [23]. While the FLAG-tagged protein evident in *P. tetraurelia* shows loss of PKD2 with depletion of *BBS7*, *BBS8*, or *BBS9*, the retention of VGCC1c is consistent with the *Chlamydomonas* results. Clearly there are species and cargo differences which probably provide insights into their particular mechanism of trafficking to cilia or IFT processes for import or retention in cilia.

It has been shown in mammalian cells that PKD2 traffics from the endoplasmic reticulum to the Golgi apparatus and on to destinations in both plasma and ciliary membranes [28]. Vesicles with PKD2 bound for cilia leave in the cis-Golgi while those bound for the plasma membrane leave the trans-Golgi [28]. The traffic of PKD2 to the primary cilium is dependent upon interaction with the BBSome [28], probably at the plasma membrane where proteins can move laterally and their sorting signals can be recognized by the BBSome [16]. The BBSomes subsequently assemble into a coat at the plasma membrane, clustering their cargoes for movement through the peri-ciliary diffusion barrier between plasma and ciliary membrane [16].

**Figure 8:**
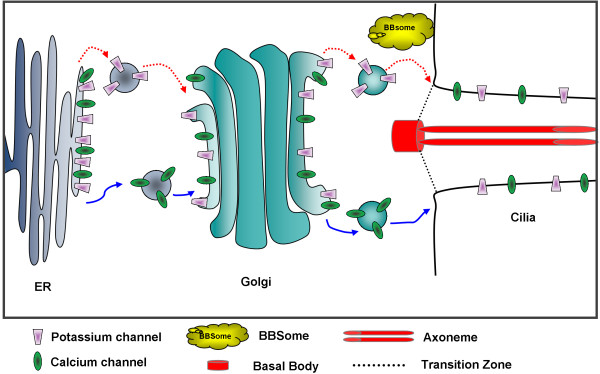
**Cartoon of BBSome trafficking in *Paramecium*. **Proteins are sorted from the endoplasmic reticulum (ER) to the Golgi and from the trans-Golgi network to the base of the cilia either in a BBSome-dependent pathway (red arrow with a dotted line) or a BBSome-independent pathway (blue arrow). The K^+^ channel (purple trapezoid) is trafficked to the cilia via the BBSome-dependent pathway while the Ca_v_ channel (green oval) is sorted via the BBSome-independent pathway. Structural proteins (not shown) traffic through cargo interactions with the BBSome to the cilia.

Taking into consideration the mammalian BBSome and ciliary membrane trafficking, and our results, we propose that the BBS proteins of the putative *Paramecium* BBS complex and BBS3 interact with protein cargo of the Golgi vesicles and/or plasma membrane that have proteins destined for the ciliary membrane (Figure 8). Furthermore, to account for the loss of cilia in RNAi for *BBS7* and *BBS9,* we propose that structural proteins can also be cargo that is assisted specifically by the BBS7 and BBS9 proteins to reach the IFT of the cilia. Whether the structural proteins for cilia development and maintenance are attached to vesicles or otherwise associate with the BBS7, BBS8 and BBS9 proteins, we cannot say. The Golgi vesicles or plasma membrane that associate with BBS7, BBS8 and BBS9 probably do not include all lipid rafts, because the folate chemoreceptor, which should reside in a raft as a glycosylphosphatidylinositol (GPI)-anchored protein, is retained in cilia in the RNAi treatments. Some transmembrane protein channels, such as VGCC1c, must be sorted to a different set of Golgi vesicles or sites in the plasma membrane other than the ones that require BBS7, BBS8 or BBS9 for incorporation into the ciliary membrane.

The BBSome of mammals appears to interact directly with ciliary targeting signals of the membrane protein cargo [16]. Ciliary targeting signals have been identified for the somatostatin receptor, rhodopsin, and fibrocystin [18,43,44]. Interestingly, the ciliary membrane proteins SK1a and PKD2 that are affected by *BBS* RNAi in *P. tetraurelia* do not carry these ciliary localization amino acid motifs. In contrast, the VGCC1c has three such mammalian motifs but is not affected by *BBS* RNAi, perhaps reflecting species differences in trafficking. Clearly, the *Paramecium* ciliary targeting signal has yet to be identified.

Not accounted for in the model in Figure 8 is that depletions of single *P. tetraurelia BBS* gene products do not all produce the same phenotype, which is not unexpected given the variety of phenotypes of BBS knockout organisms [45-47]. It apparently takes multiple BBS mutations to produce the full syndrome in humans [48], meaning that the BBSome may have partial function when missing a component or that the BBS proteins have functions independent of the BBSome.

In our model, the loss of cilia from the *BBS7*- and *BBS9*-depleted cells arises from the BBS proteins failing to transport structure proteins as cargo. Alternatively, as Jin *et al.*, [16] speculate, interference with the BBSome results in reduced ciliary length due to interference with loading the IFT. In *P. tetraurelia*, length differences are seen with *BBS7* and *BBS9* RNAi, while in mammals reduced ciliary length and numbers are associated with *BBS1* and *BBS4*. The exception to the *P. tetraurelia* BBS phenotype described here is *BBS2* that does not appear to be obligatory for the location of K^+^ channels in cilia, as we might have expected from its role in trafficking of rhodopsin in retinal cells [45]. Also, ciliary morphology or number does not suffer from reduction of *BBS2* or *BBS4* in *P. tetraurelia* as it does in airway cilia [6]. These species differences are important to note because they can eventually provide insights into interactions and capabilities of the BBS proteins that cannot be derived from study of a single model organism.

Sensory function of cilia in other organisms also involves ion channels. For example, *Chlamydomonas* cells express in their flagella a voltage-gated calcium channel that is necessary for the change in wave form in response to light or mechanical stimulation [10]. The cilia of olfactory sensory neurons have cyclic nucleotide-gated non-specific cation channels [49], calcium-dependent K^+^ channels [50-52] and calcium-dependent Cl^-^ channels [53], all of which contribute to the olfactory sensory function of these cilia. PKD2, a member of the transient receptor potential protein family of non-specific cation channels, is found in primary cilia of mammalian epithelial cell types; PKD2 conducts Ca^2+^ among other ions and functions as a mechanoreceptor [54-58]. The CatSper calcium channels of the sperm flagellum are responsible for the change in wave form in the vicinity of the egg [59,60].

## Conclusions

By using *P. tetraurelia*, we focus on the ion channels that govern ciliary motility as well as sensory function. We combine this focus with depletion of ciliopathy gene products, in this case BBS genes. Our results show that in *P. tetraurelia* there is selection among the ion channels that govern ciliary motility and sensory function in their dependence upon BBS proteins to reach or remain in the ciliary membrane. We can efficiently combine RNAi reduction of BBS and other ciliopathy gene products with the swimming behavior read-out of ciliary channel and other membrane protein function, making good use of a model organism that has a rich history of research on its ciliary motility and physiology.

## Abbreviations

AP: Alkaline phosphatase; BBS: Bardet-Biedl syndrome; BME: β-mercaptoethanol; Ca_v_: Voltage-dependent calcium channel; CTS: Cilia targeting signal; dCn2: Unique delta correlation score; DIC: Differential interference contrast; EGTA: Ethylene glycol tetraacetic acid; ER: Endoplasmic reticulum; FBP: Folate binding protein; GPI: Glycosylphosphatidylinositol; HEPES: Hydroxyethyl piperazineethanesulfonic acid; HRP: Horseradish peroxidase; HPLC: High performance liquid chromatography; IFT: Intraflagellar transport; IP: Immunoprecipitation; K_Ca_: Calcium-dependent potassium channel; K_V_: Voltage-dependent potassium channel; I_Ca_: Voltage-dependent calcium current; I_KCa_: Calcium-dependent potassium current; I_KV_: Voltage-dependent potassium current; KCl: Potassium chloride; MS: Mass spectrometry; PBS: Phosphate-buffered saline; PIPES: Piperazine ethanesulfonic acid; PKD2: Polycystin-2; RT: Room temperature; RT-PCR: Reverse transcription polymerase chain reaction; SK1a: Small conductance potassium channel; SEM: Standard error of the mean; TEA: Tetraethylammonium chloride; VGCC1c: Voltage-dependent calcium channel isoform 1c; WCE: Whole cell extract.

## Competing interests

The authors declare that they have no competing interests.

## Authors’ contributions

MSV: Cloning and preparation of PKD2 constructs, demonstration that PKD2 is localized in cilia, all experimental results and data analysis for BBS2, manuscript preparation, editing, and submission, all figure preparation; AR: construct construction of RNAi and expression vectors for BBS7 and BBS9, immunofluorescence, mass spectrometry, behavioral analysis, data collection; JY: voltage-dependent calcium channel constructs and IP with RNAi data, significant contribution to experiment design, critical reading of manuscript; SDW: folate chemoreceptor antibody preparation, development of RNAi vector for the chemoreceptor, demonstration of chemoreceptor in cilia, critical reading of manuscript; JB, JC, and FK: construct design and creation for BBS1, 2, 3, 4, 5 and 8, critical reading of manuscript and assistance with experimental guidance and design; JVH: writing, critical reading and editing of manuscript, principal investigator of the lab where all experiments took place, experimental guidance and assistance. All authors read and approved the final manuscript.

## Supplementary Material

Additional file 1 **Table S1. **Paramecium BBS sequences compared to Human *BBS *sequences. Click here for file

Additional file 2 **Figure S1. **Representative results of semi-quantitative RT-PCR to evaluate the endogenous level of mRNA in RNAi treated cells. PCR amplification of serially diluted cDNA from *BBS8*-depleted cells (test) and control cells (control) using the RT-PCR primers for *BBS8 *transcript. As a template control, serially diluted cDNA from the test and control cells were amplified using calmodulin gene primers. In both gels, the dilutions are listed below the lanes. Approximate sizes of the bands are to the left of the image. Lane labeled -RT is a negative control containing cDNA prepared without reverse transcriptase. No band is present indicating no genomic DNA contamination. Click here for file

Additional file 3 **Figure S2. **Silver stained gels and peptide distribution histograms for FLAG-BBS9 and FLAG-BBS8 immunoprecipitation and Mass spec analysis. Whole cell extracts were isolated from cells expressing empty FLAG vector and *FLAG-BBS9 ***(A) **or *FLAG-BBS8 ***(B)**, immunoprecipitated using anti-FLAG affinity beads and separated on a 7 to 18% SDS-PAGE gel and silver stained. Eight segments of the gels (S1-S8) were removed and subjected to a trypsin digest and mass spectrometry analysis (see Materials and Methods). To the right of each gel is a histogram depicting the different BBS protein peptides which immunoprecipitated with BBS9 **(A) **and BBS8 **(B) **and the segment of the gel in which the unique peptide was identified. Non-BBS proteins that were identified can be seen in Additional file 4: Table S2. and Additional file 5: Table S3, respectively. All members of the mammalian BBSome were identified from the FLAG-BBS9 IP (BBS1, BBS2, BBS4, BBS5, BBS7, BBS8, and BBS9). All but BBS4 were identified from the FLAG-BBS8 IP. Click here for file

Additional file 4 **Table S2. **List of non-BBS proteins immunoprecipitated with FLAG-BBS9. Click here for file

Additional file 5 **Table S3. **List of non-BBS proteins immunoprecipitated with FLAG-BBS8. Click here for file

Additional file 6 **Figure S3. **Combined depletion of BBS and the Folate chemoreceptor (FBP) in cells expressing FLAG-SK1a. Cells were immunostained for the FLAG-SK1a channel (anti-FLAG; red) and the folate chemoreceptor (anti-FBP; green). Negative control cells expressing the empty FLAG vector fed the RNAi empty vector (L4440) are shown in the top row indicating clear FBP staining. Positive control cells expressing *FLAG-SK1a* were fed RNAi empty vector and show clear FLAG (red) and FBP (green) staining. Cells expressing *FLAG-SK1a* were fed a combination of RNAi for *FBP* and *BBS7, BBS8* or *BBS9*. Note the extensive loss of the FBP protein with RNAi. Click here for file

Additional file 7 **Figure S4. **Percentage of ciliated and bald cells for *BBS7-, BBS8-,* and *BBS9*-depleted cells. The cells observed were expressing *FLAG-SK1a* and fed RNAi for *BBS7*, *BBS8* or *BBS9.* These cells were observed using DIC and were scored as being bald (> 75% deciliated) or ciliated. Observations were pooled from three separate experiments, n = 61 to 71 cells. Click here for file

Additional file 8 **Table S4. **Backward Swimming in 8 mM BaCl_2_. Data are ± standard deviation (SD) normalized to the control for time spent backward swimming. N represents the number of cells tested. Mann–Whitney *U*-test determined no significant differences. Click here for file

Additional file 9 **Figure S5. **Immunofluorescence of cells expressing the *FLAG-SK1a* channel fed RNAi for *BBS7*, *BBS8*, or *BBS9*. Cells were immunostained for the folate chemoreceptor (anti-FBP; green) and FLAG-SK1a (anti-FLAG; red). Control cells expressing the empty FLAG vector were fed the RNAi empty vector (L4440) bacteria. The control FLAG-SK1a cells were fed the RNAi empty vector (L4440). FLAG-SK1a cells were fed the RNAi for *BBS7*, *BBS8* and *BBS9.* Images were taken under 60× oil immersion objectives. Scales represent 15 μm. Images are representative of results of three experiments, n =126 to 156 cells. The DIC images are shown to demonstrate that cilia are still present on these cells. Click here for file

Additional file 10 **Figure S6. **Fluorescence images of cells expressing *PKD2-FLAG* channel and also are *BBS7*-, *BBS8*- or *BBS9*-depleted by RNAi. Control cells expressing the empty FLAG vector were fed the RNAi empty vector (L4440). *PKD2-FLAG* channel-expressing cells were fed the RNAi empty vector control (L4440), followed by *PKD2-FLAG* channel-expressing cells fed RNAi for *BBS7*, *BBS8* or *BBS9.* Cells were immunostained with anti-FLAG (red) and anti-centrin-1 (green) antibodies. Images were taken under 60× oil immersion objectives. Scales represent 15 μm. Images are representative of results of three experiments, n = 126 to 156 cells. DIC images show that cilia are present. Click here for file

Additional file 11 **Figure S7. **Backward swimming duration after stimulation with TEA and High Na^+^ or TEA with High Mg^2+^. Before cells were used in experiments seen in Figure 6 and Additional file 9: Figure S5. and Additional file 10: Figure S6. respectively, they were tested for their swimming in TEA solutions with Na^+^ and Mg^2+^, which was diagnostic for the successful effects of RNAi for *BBS7*, *BBS8* and *BBS9*. Data were normalized to the control backward swimming duration. Pairs of graphs relate to each experiment: **(A)** and **(B)** to FLAG-SK1a Figure 6 and Additional file 9: Figure S5; **(C)** and **(D)** to PKD2-FLAG Figure 6 and Additional file 10: Figure S6.. **denotes significant difference from normalized control using the Mann–Whitney *U*-test (*P* < 0.0001). Data are averages from 60 cells ± standard error of the mean (SEM) for the behavioral tests in Na^+^ with TEA and Mg^2+^ with TEA tests. Click here for file
