# Supplementary material for: Paramecium BBS genes are key to presence of channels in Cilia
Source: Cilia. 2012 Sep 3;1:16. doi: 10.1186/2046-2530-1-16 (PMC3556005; doi:10.1186/2046-2530-1-16)
Supplement: Additional file 1 — Table S1. Paramecium BBS sequences compared to Human BBS sequences. [file 2046-2530-1-16-S1.docx]

**Additional file: Table 1.** *Paramecium* *BBS* sequences compared to Human *BBS* sequences.

| **Gene Name** | **(GSPATG…)^a^** | **HA #^b^** | **e^- c^** | **% AAI^d^** | **RNAi^e^** |
| --- | --- | --- | --- | --- | --- |
| *BBS1* | 00033252001 | NP_078925 | 4 e^-57^ | 26 | +1 - 1724 |
| *BBS2* | 00000964001 | BAF82293 | 1 e^-61^ | 26 | +1377 - +1935 |
| *BBS3a/ARL_B64* | 00017502001 | NP_115522 | 3 e^-28^ | 43 | +1 - 649 |
| *BBS3b/ARL_A64* | 00038302001 | NP_115522 | 2 e^-29^ | 44 | +1 - 603 |
| *BBS4* | 00005292001 | AAS13441 | 3 e^-57^ | 36 | +1 - 1349 |
| *BBS5a* | 00036912001 | NP_689597 | 1 e^-43^ | 30 | +1 - 981 |
| *BBS5.b* | 00034169001 | NP_689597 | 4 e^-41^ | 30 | +1 - 981 |
| *BBS7* | 00026091001 | NP_789794 | 4 e^-40^ | 25 | +102 -1836 |
| *BBS8* | 00028481001 | AAR19043 | 1 e^-104^ | 37 | +1 - 1626 |
| *BBS9* | 00027545001 | AAH32715 | 1 e^-29^ | 33 | +142 - 1869 |

^a^ The *Paramecium* gene accession number which begins with GSPATG.

^b^ The accession number for the human gene (HA#) located from BLAST searches with the *Paramecium* gene.

^c^ The corresponding e- value from the BLAST search.

^d^ The percent amino acid identity (%AAI) between the human and *Paramecium* proteins sequences.

^e^ The positions of the *BBS* RNAi construct within the gene.
