# Supplementary figures and images for: Paramecium BBS genes are key to presence of channels in Cilia
Source: Cilia. 2012 Sep 3;1:16. doi: 10.1186/2046-2530-1-16 (PMC3556005; doi:10.1186/2046-2530-1-16)

### BBS8 specific primers

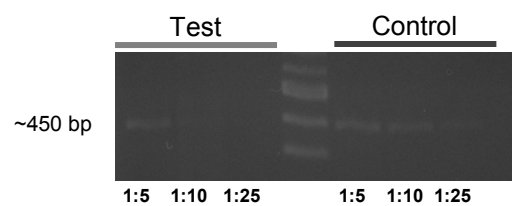

### Calmodulin specific primers

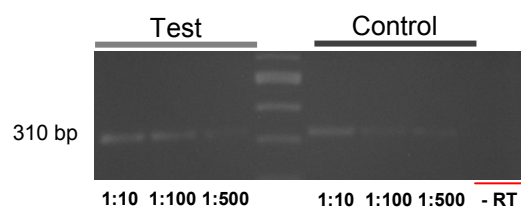

Supplement: Additional file 2 — Figure S1. Representative results of semi-quantitative RT-PCR to evaluate the endogenous level of mRNA in RNAi treated cells. PCR amplification of serially diluted cDNA from BBS8-depleted cells (test) and control cells (control) using the RT-PCR primers for BBS8 transcript. As a template control, serially diluted cDNA from the test and control cells were amplified using calmodulin gene primers. In both gels, the dilutions are listed below the lanes. Approximate sizes of the bands are to the left of the image. Lane labeled -RT is a negative control containing cDNA prepared without reverse transcriptase. No band is present indicating no genomic DNA contamination. [file 2046-2530-1-16-S2.pdf]

**A. FLAG-BBS9 Immunoprecipitation**

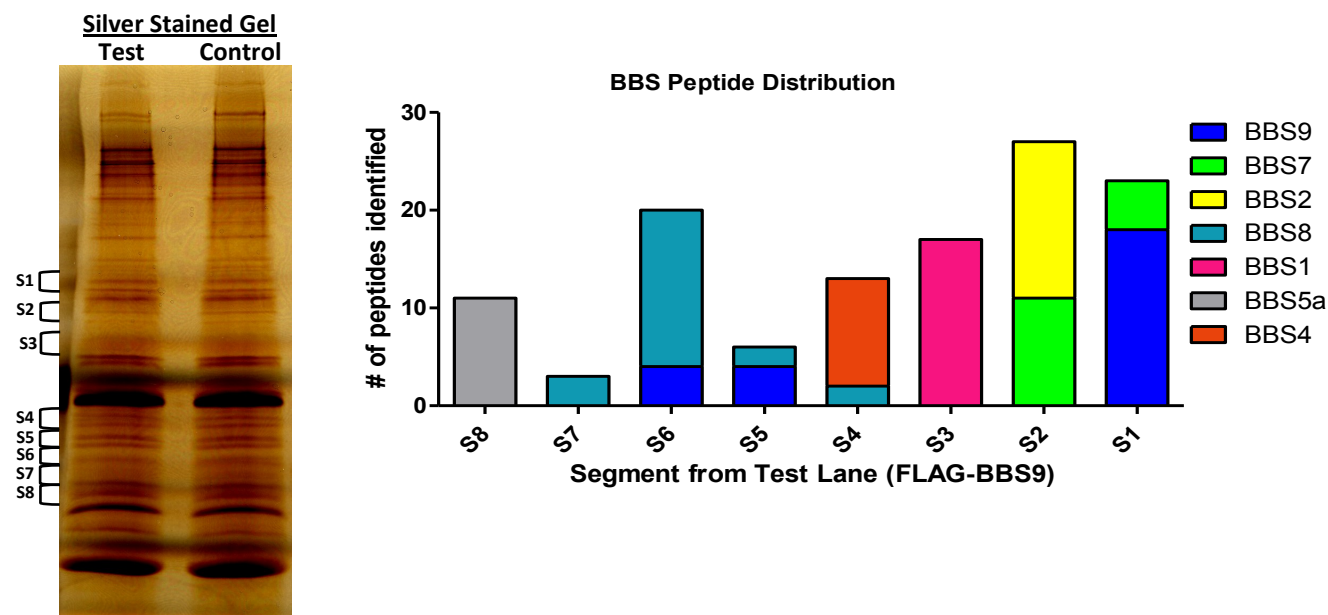

**B. FLAG-BBS8 Immunoprecipitation**

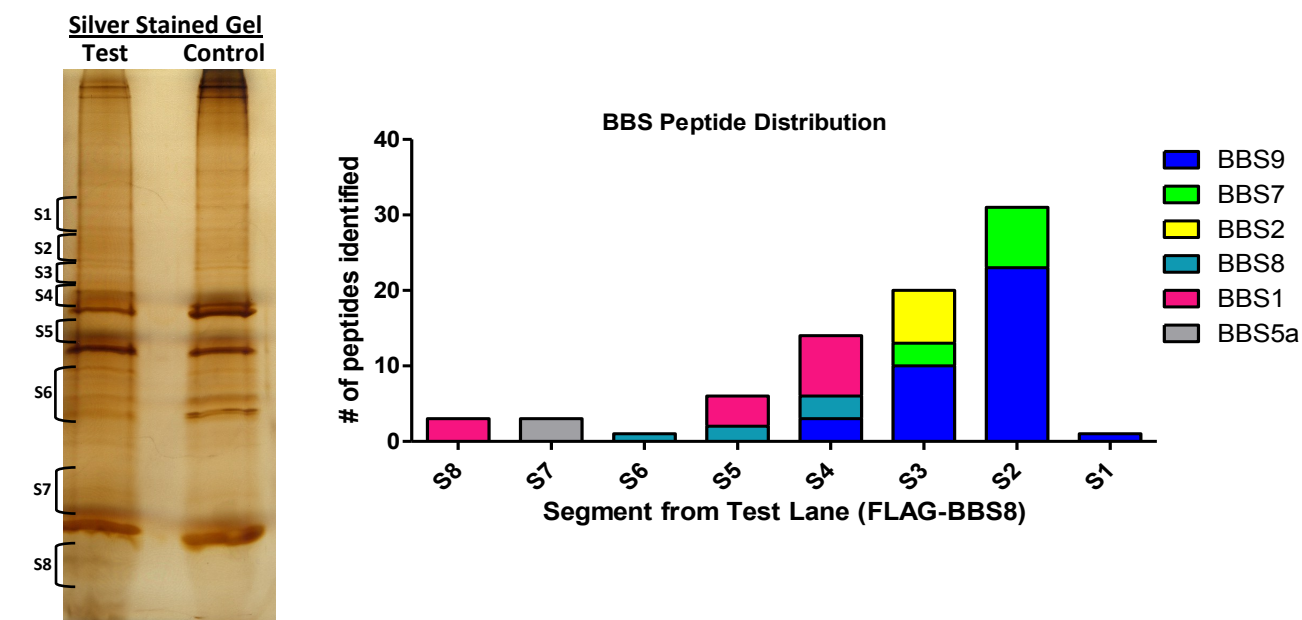

Supplement: Additional file 3 — Figure S2. Silver stained gels and peptide distribution histograms for FLAG-BBS9 and FLAG-BBS8 immunoprecipitation and Mass spec analysis. Whole cell extracts were isolated from cells expressing empty FLAG vector and FLAG-BBS9 (A) or FLAG-BBS8 (B), immunoprecipitated using anti-FLAG affinity beads and separated on a 7 to 18% SDS-PAGE gel and silver stained. Eight segments of the gels (S1-S8) were removed and subjected to a trypsin digest and mass spectrometry analysis (see Materials and Methods). To the right of each gel is a histogram depicting the different BBS protein peptides which immunoprecipitated with BBS9 (A) and BBS8 (B) and the segment of the gel in which the unique peptide was identified. Non-BBS proteins that were identified can be seen in Additional file 4: Table S2. and Additional file 5: Table S3, respectively. All members of the mammalian BBSome were identified from the FLAG-BBS9 IP (BBS1, BBS2, BBS4, BBS5, BBS7, BBS8, and BBS9). All but BBS4 were identified from the FLAG-BBS8 IP. [file 2046-2530-1-16-S3.pdf]

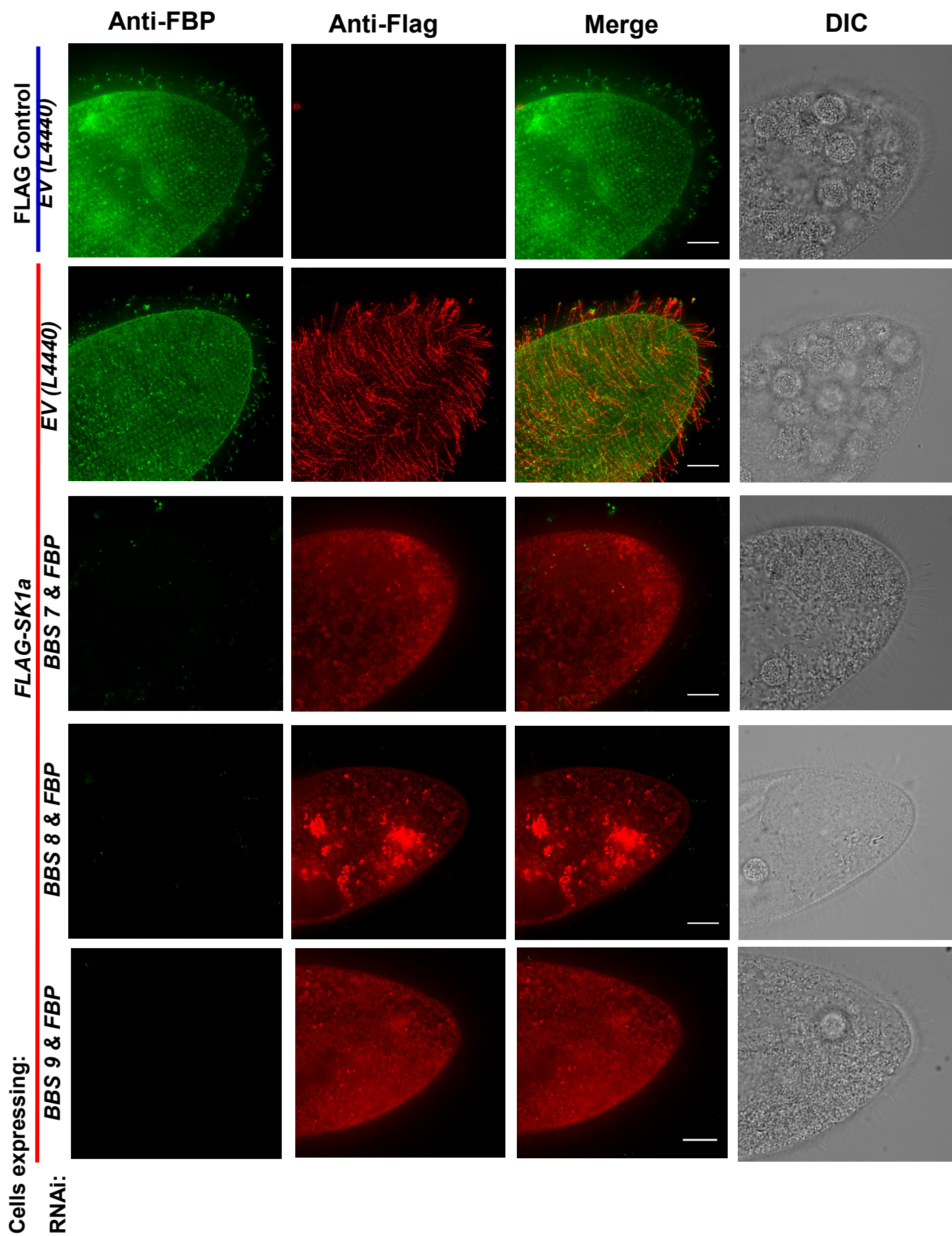

Supplement: Additional file 6 — Figure S3. Combined depletion of BBS and the Folate chemoreceptor (FBP) in cells expressing FLAG-SK1a. Cells were immunostained for the FLAG-SK1a channel (anti-FLAG; red) and the folate chemoreceptor (anti-FBP; green). Negative control cells expressing the empty FLAG vector fed the RNAi empty vector (L4440) are shown in the top row indicating clear FBP staining. Positive control cells expressing FLAG-SK1a were fed RNAi empty vector and show clear FLAG (red) and FBP (green) staining. Cells expressing FLAG-SK1a were fed a combination of RNAi for FBP and BBS7, BBS8 or BBS9. Note the extensive loss of the FBP protein with RNAi. [file 2046-2530-1-16-S6.pdf]

**BBS7**

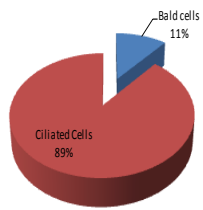

**BBS8**

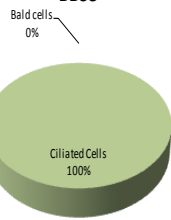

**BBS9**

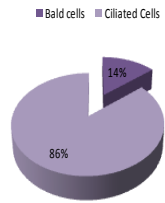

Supplement: Additional file 7 — Figure S4. Percentage of ciliated and bald cells for BBS7-, BBS8-, and BBS9-depleted cells. The cells observed were expressing FLAG-SK1a and fed RNAi for BBS7, BBS8 or BBS9. These cells were observed using DIC and were scored as being bald (> 75% deciliated) or ciliated. Observations were pooled from three separate experiments, n = 61 to 71 cells. [file 2046-2530-1-16-S7.pdf]

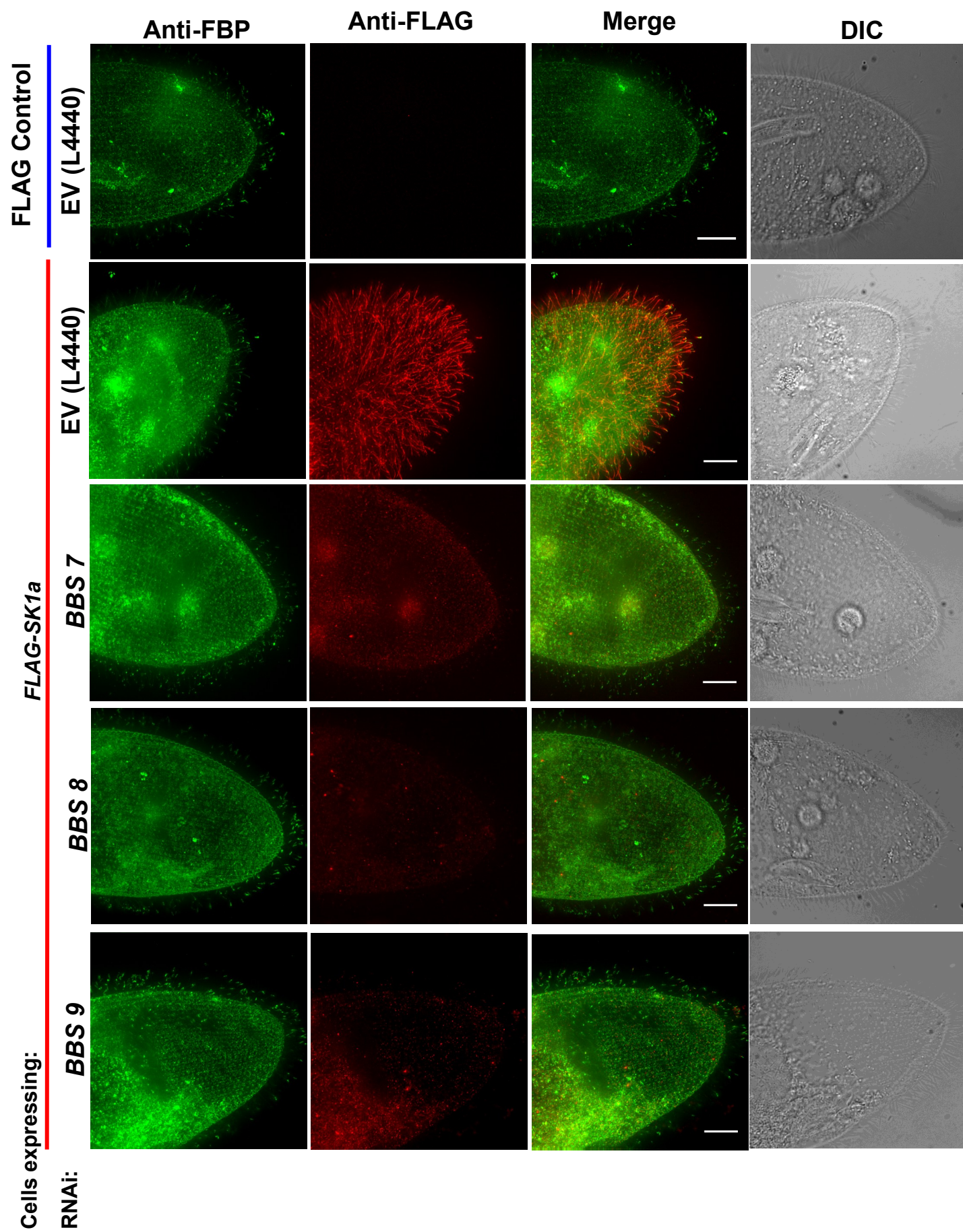

Supplement: Additional file 9 — Figure S5. Immunofluorescence of cells expressing the FLAG-SK1a channel fed RNAi for BBS7, BBS8, or BBS9. Cells were immunostained for the folate chemoreceptor (anti-FBP; green) and FLAG-SK1a (anti-FLAG; red). Control cells expressing the empty FLAG vector were fed the RNAi empty vector (L4440) bacteria. The control FLAG-SK1a cells were fed the RNAi empty vector (L4440). FLAG-SK1a cells were fed the RNAi for BBS7, BBS8 and BBS9. Images were taken under 60× oil immersion objectives. Scales represent 15 μm. Images are representative of results of three experiments, n =126 to 156 cells. The DIC images are shown to demonstrate that cilia are still present on these cells. [file 2046-2530-1-16-S9.pdf]

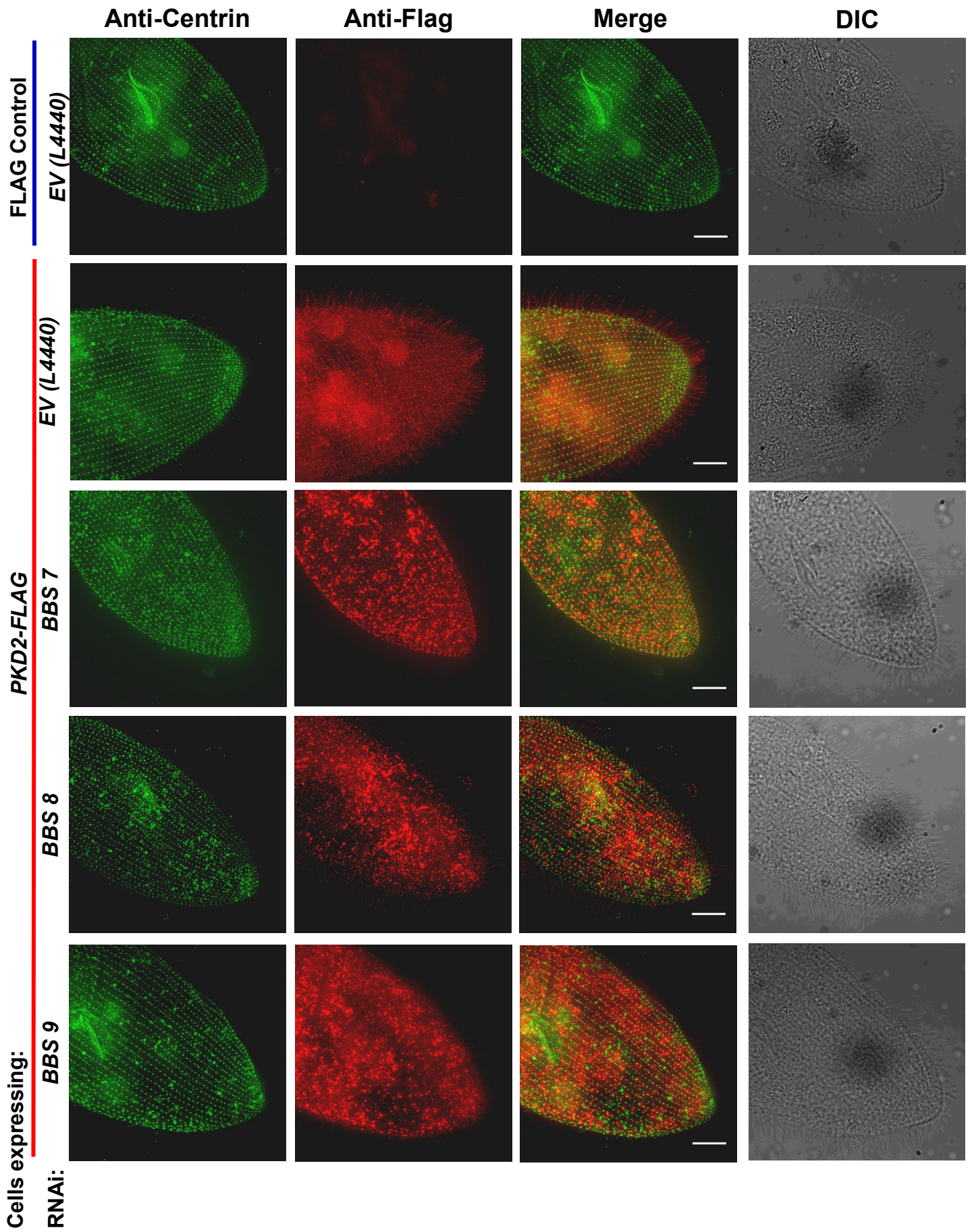

Supplement: Additional file 10 — Figure S6. Fluorescence images of cells expressing PKD2-FLAG channel and also are BBS7-, BBS8- or BBS9-depleted by RNAi. Control cells expressing the empty FLAG vector were fed the RNAi empty vector (L4440). PKD2-FLAG channel-expressing cells were fed the RNAi empty vector control (L4440), followed by PKD2-FLAG channel-expressing cells fed RNAi for BBS7, BBS8 or BBS9. Cells were immunostained with anti-FLAG (red) and anti-centrin-1 (green) antibodies. Images were taken under 60× oil immersion objectives. Scales represent 15 μm. Images are representative of results of three experiments, n = 126 to 156 cells. DIC images show that cilia are present. [file 2046-2530-1-16-S10.pdf]
