# Supplementary material for: Paramecium BBS genes are key to presence of channels in Cilia
Source: Cilia. 2012 Sep 3;1:16. doi: 10.1186/2046-2530-1-16 (PMC3556005; doi:10.1186/2046-2530-1-16)
Supplement: Additional file 4 — Table S2. List of non-BBS proteins immunoprecipitated with FLAG-BBS9. [file 2046-2530-1-16-S4.docx]

**Additional file: Table S2.** **List of non-BBS proteins immunoprecipitated with FLAG-BBS9**

Total numbers of unique peptides for each protein that were found exclusively in the FLAG-BBS9 lane are summarized. The annotation number and functional domain for each protein is as described in the *Paramecium* genome.

| **Annotation #** | **Protein Name/ functional Domain** | **FLAG-BBS9** | | **Control** |
| --- | --- | --- | --- | --- |
|  |  | **Unique** | **Total** |  |
| **GSPATP00038477001** | Peptidase C1A, papain | 5 | 9 | 0 |
| **GSPATP00025676001** | Glucosamine-6-phosphate isomerase | 5 | 7 | 0 |
| **GSPATP00028551001** | Trichocyst matrix protein, putative | 3 | 3 | 0 |
| **GSPATP00036466001** | Armadillo-type fold | 3 | 3 | 0 |
| **GSPATP00009104001** | Spliceosome RNA helicase BAT1 | 3 | 3 | 0 |
| **GSPATP00012116001** | Serine/threonine-specific protein phosphatase | 3 | 3 | 0 |
| **GSPATP00010403001** | WD40 repeat | 3 | 3 | 0 |
| **GSPATP00007051001** | ATP-dependent protease La | 2 | 3 | 0 |
| **GSPATP00031501001** | Ectonucleoside triphosphate diphosphohydrolase 1 | 2 | 3 | 0 |
| **GSPATP00032991001** | ADP/ATP translocase 4 | 2 | 3 | 0 |
| **GSPATP00017574001** | calcium-dependent membrane targeting | 2 | 2 | 0 |
| **GSPATP00034004001** | K(V) β subunits | 2 | 2 | 0 |
| **GSPATP00020011001** | Hypothetical Protein | 2 | 2 | 0 |
| **GSPATP00037101001** | Centrin-1 | 2 | 2 | 0 |
| **GSPATP00013517001** | MCM | 2 | 2 | 0 |
| **GSPATP00007464001** | Non-specific lipid-transfer protein | 2 | 2 | 0 |
| **GSPATP00008233001** | THO complex subunit 1 | 2 | 2 | 0 |
| **GSPATP00004209001** | Nucleotide-binding, alpha-beta plait | 2 | 2 | 0 |
