# Supplementary material for: Paramecium BBS genes are key to presence of channels in Cilia
Source: Cilia. 2012 Sep 3;1:16. doi: 10.1186/2046-2530-1-16 (PMC3556005; doi:10.1186/2046-2530-1-16)
Supplement: Additional file 5 — Table S3. List of non-BBS proteins immunoprecipitated with FLAG-BBS8. [file 2046-2530-1-16-S5.docx]

**Additional file: Table S3. List of non-BBS proteins immunoprecipitated with FLAG-BBS8**

Total numbers of unique peptides for each protein that were found exclusively in the FLAG-BBS8 lane are summarized. The annotation number and functional domain for each protein is as described in the *Paramecium* genome is included.

| **Annotation #** | **Protein Name/ functional Domain** | **FLAG-BBS8** | | **Control** |
| --- | --- | --- | --- | --- |
|  |  | **Unique** | **Total** |  |
| **GSPATP00010708001** | Mitochondrial Ribosomal protein s29, putative | 2 | 2 | 0 |
| **GSPATP00005547001** | Ribosomal protein L40e | 3 | 5 | 0 |
