# Supplementary material for: Paramecium BBS genes are key to presence of channels in Cilia
Source: Cilia. 2012 Sep 3;1:16. doi: 10.1186/2046-2530-1-16 (PMC3556005; doi:10.1186/2046-2530-1-16)
Supplement: Additional file 8 — Table S4. Backward Swimming in 8 mM BaCl2. Data are ± standard deviation (SD) normalized to the control for time spent backward swimming. N represents the number of cells tested. Mann–Whitney U-test determined no significant differences. [file 2046-2530-1-16-S8.docx]

**Additional file: Table S4.** Backward Swimming in 8 mM BaCl_2_. Data are ± standard deviation (SD) normalized to the control for time spend backward swimming. N represents the number of cells tested. Mann-Whitney U test determined no significant differences.

| **Cell line** | **8 mM BaCl_2_**  **(normalized ± SD, N= # of cells)** |
| --- | --- |
| **Control** | 100.0% ± 65.4% (N=45) |
| ***BBS1*** | 104.4% ± 80.9% (N=90) |
| ***BBS2*** | 106.0% ± 99.6% (N=120) |
| ***BBS3*** | 115.9% ± 46.3% (N=45) |
| ***BBS4*** | 98.9% ± 49.3% (N=45) |
| ***BBS5*** | 105.7% ± 43.8% (N=45) |
| ***BBS7*** | 94.9% ± 50.4% (N=45) |
| ***BBS8*** | 84.3% ± 59.3% (N=90) |
| ***BBS9*** | 82.1% ± 45.6% (N=90) |
