# Supplementary material for: Paramecium BBS genes are key to presence of channels in Cilia
Source: Cilia. 2012 Sep 3;1:16. doi: 10.1186/2046-2530-1-16 (PMC3556005; doi:10.1186/2046-2530-1-16)
Supplement: Additional file 11 — Figure S7. Backward swimming duration after stimulation with TEA and High Na+ or TEA with High Mg2+. Before cells were used in experiments seen in Figure 6 and Additional file 9: Figure S5. and Additional file 10: Figure S6. respectively, they were tested for their swimming in TEA solutions with Na+ and Mg2+, which was diagnostic for the successful effects of RNAi for BBS7, BBS8 and BBS9. Data were normalized to the control backward swimming duration. Pairs of graphs relate to each experiment: (A) and (B) to FLAG-SK1a Figure 6 and Additional file 9: Figure S5; (C) and (D) to PKD2-FLAG Figure 6 and Additional file 10: Figure S6.. **denotes significant difference from normalized control using the Mann–Whitney U-test (P < 0.0001). Data are averages from 60 cells ± standard error of the mean (SEM) for the behavioral tests in Na+ with TEA and Mg2+ with TEA tests. [file 2046-2530-1-16-S11.pdf]

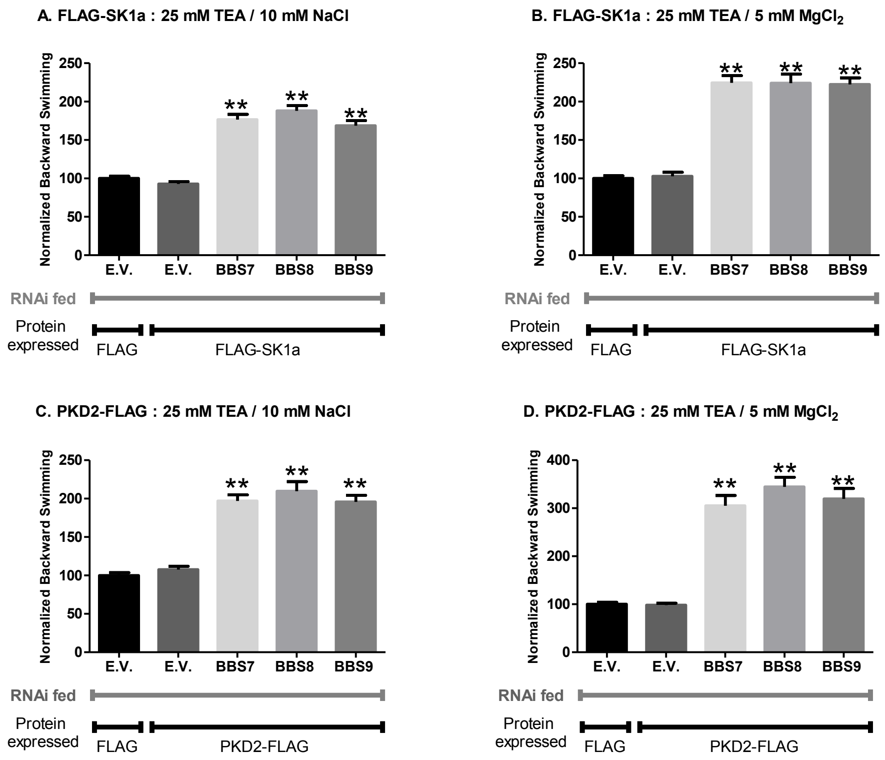
A. FLAG-SK1a : 25 mM TEA / 10 mM NaCl
B. FLAG-SK1a : 25 mM TEA / 5 mM MgCl2
C. PKD2-FLAG : 25 mM TEA / 10 mM NaCl
D. PKD2-FLAG : 25 mM TEA / 5 mM MgCl2
Normalized Backward Swimming
0
50
100
150
200
250
300
400
E.V.
E.V.
BBS7
BBS8
BBS9
**
RNAi fed
Protein expressed
FLAG
FLAG-SK1a
PKD2-FLAG
